# Ligand Engineering in Tin-Based Perovskite Solar Cells

**DOI:** 10.1007/s40820-023-01143-0

**Published:** 2023-07-03

**Authors:** Peizhou Li, Xiangrong Cao, Jingrui Li, Bo Jiao, Xun Hou, Feng Hao, Zhijun Ning, Zuqiang Bian, Jun Xi, Liming Ding, Zhaoxin Wu, Hua Dong

**Affiliations:** 1 Key Laboratory for Physical Electronics and Devices (MoE), Shaanxi Key Lab of Information Photonic Technique, School of Electronic and Information Engineering, Xi’an Jiaotong University, Xi’an, 710049 People’s Republic of China; 2 Center for Excellence in Nanoscience (CAS), Key Laboratory of Nanosystem and Hierarchical Fabrication (CAS), National Center for Nanoscience and Technology, Beijing, 100190 People’s Republic of China; 3 School of Materials and Energy, University of Electronic Science and Technology of China, Chengdu, 611731 People’s Republic of China; 4 School of Physical Science and Technology, ShanghaiTech University, Shanghai, 201210 People’s Republic of China; 5 Beijing National Laboratory for Molecular Sciences, State Key Laboratory of Rare Earth Materials Chemistry and Applications, College of Chemistry and Molecular Engineering, Peking University, Beijing, 100871 People’s Republic of China; 6 Collaborative Innovation Center of Extreme Optics, Shanxi University, Taiyuan, 030006 People’s Republic of China

**Keywords:** Perovskite, Solar cells, Lead-free, Ligand engineering, Defects, Stability

## Abstract

Perovskite solar cells (PSCs) have attracted aggressive attention in the photovoltaic field in light of the rapid increasing power conversion efficiency. However, their large-scale application and commercialization are limited by the toxicity issue of lead (Pb). Among all the lead-free perovskites, tin (Sn)-based perovskites have shown potential due to their low toxicity, ideal bandgap structure, high carrier mobility, and long hot carrier lifetime. Great progress of Sn-based PSCs has been realized in recent years, and the certified efficiency has now reached over 14%. Nevertheless, this record still falls far behind the theoretical calculations. This is likely due to the uncontrolled nucleation states and pronounced Sn (IV) vacancies. With insights into the methodologies resolving both issues, ligand engineering-assisted perovskite film fabrication dictates the state-of-the-art Sn-based PSCs. Herein, we summarize the role of ligand engineering during each state of film fabrication, ranging from the starting precursors to the ending fabricated bulks. The incorporation of ligands to suppress Sn^2+^ oxidation, passivate bulk defects, optimize crystal orientation, and improve stability is discussed, respectively. Finally, the remained challenges and perspectives toward advancing the performance of Sn-based PSCs are presented. We expect this review can draw a clear roadmap to facilitate Sn-based PSCs via ligand engineering.

## Highlights

- Systematic summary of ligand engineering in Sn-based perovskite solar cells at the molecular level (oxidation-suppression), crystal structural level (bulk-defect passivation and crystal orientation optimization), and film level (film stability).
- The classification and composition of ligand engineering in the review are the same as the actual preparation process, which will help researchers to understand the role of ligands in combination with the actual experiment process.
- Description of ligands focuses on the function of each functional group; the relevant conclusion can be universal.

## Graphical abstract

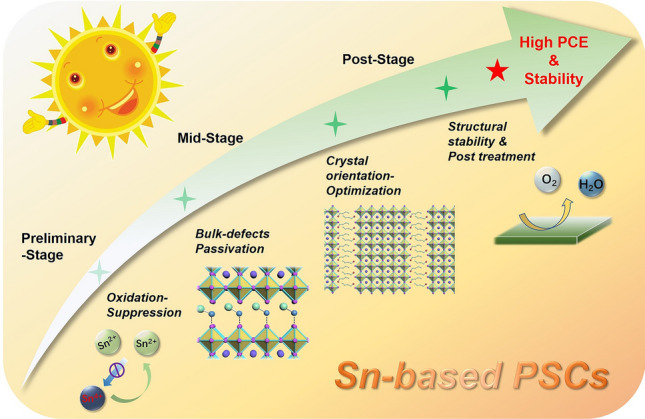

## Introduction

The past decade has witnessed the impressive progress of organic–inorganic halide perovskites in the fields of solar cells, photodetectors, and light-emitting diodes. The highest certified power conversion efficiency (PCE) of perovskite solar cells (PSCs) has reached 25.7%, accompanied by outstanding properties including effective light absorption, adjustable bandgap, long carrier diffusion length, and solution preparable process [1–3]. Despite the excellent optoelectronic properties, the toxicity of Pb remains to be a critical issue that hinders further application and commercialization [4, 5]. To deal with this concern, an increasing number of works have focused on developing lead-free perovskites. Other group IVA metals, for example, germanium (Ge) and tin (Sn) [6–9], along with group VA metals antimony (Sb) [10–12] and bismuth (Bi) [13, 14], have been proposed to substitute Pb. Among all the candidates, Sn perovskite has proved its unique potential by achieving a PCE of over 14% in a short period [15, 16]. Indeed, we would find Sn perovskite a promising material with a suitable optical bandgap of 1.2–1.4 eV, which could realize a theoretical maximum PCE of 33% [17–19]. Meanwhile, due to belonging to the same group as Pb does, Sn-based perovskites exhibit similar optoelectronic properties as Pb-based perovskites. Hence, numerous efforts have been made to investigate Sn-based perovskites and their application in the field of solar cells.

Despite the exciting characteristics, it should be noticed that the reported highest PCE of Sn-based PSCs still falls behind those Pb-based counterparts. Meanwhile, the issue of stability also hinders the way for further investigation, such as longer device lifetime, third-party certification, and large-scale application. At the current stage, the photovoltaic performance of Sn-based perovskites is mainly limited by the intrinsic properties of Sn-based perovskite materials. Sn element with *ns*^*2*^*np*^*2*^ electron structure owns a weaker inert pair effect than its analogue Pb, which leads to a strong tendency of oxidation. The break of Sn-I bonds caused by the oxidation process will result in the formation of Sn(IV) oxide compounds. It is also revealed that the oxidation process will involve multiple adjacent Sn^2+^ ions to form SnO_2_ and SnI_4_, which degrade the [SnI_6_]^4−^ unit as well as the perovskite lattice [20]. Furthermore, due to the high p orbital energy of I and strong antibonding coupling of Sn 5*s* and I 5*p* states, the formation energy of Sn vacancies is relatively lower in both Sn-rich and Sn-poor conditions, thus resulting in p-type characteristic with a high concentration of hole [21]. The easy oxidation of Sn^2+^ and the low formation energy of Sn vacancies both attribute to high density of defect states and unbalanced charge carrier transportation. Therefore, the inevitable corresponding non-radiative recombination would like to contribute to the inferior PCE, and the distortion of the lattice would lead to restricted stability. Different from Pb-based perovskites, factors that deteriorate Sn-based perovskites exist in the whole process of “source-intermediate state-post treatment” when fabricating Sn-based PSCs. The quality of perovskite film still plays the most important role in achieving Sn-based PSCs with high efficiency. So far, many studies have proven the effectiveness of ligand engineering in improving the photovoltaic performance of Sn-based PSCs. Ligand engineering and strategies, due to their diversified selection and versatile performance, could be the key to optimizing the film growth during the whole PSCs-device fabrication process.

**Fig. 1:**
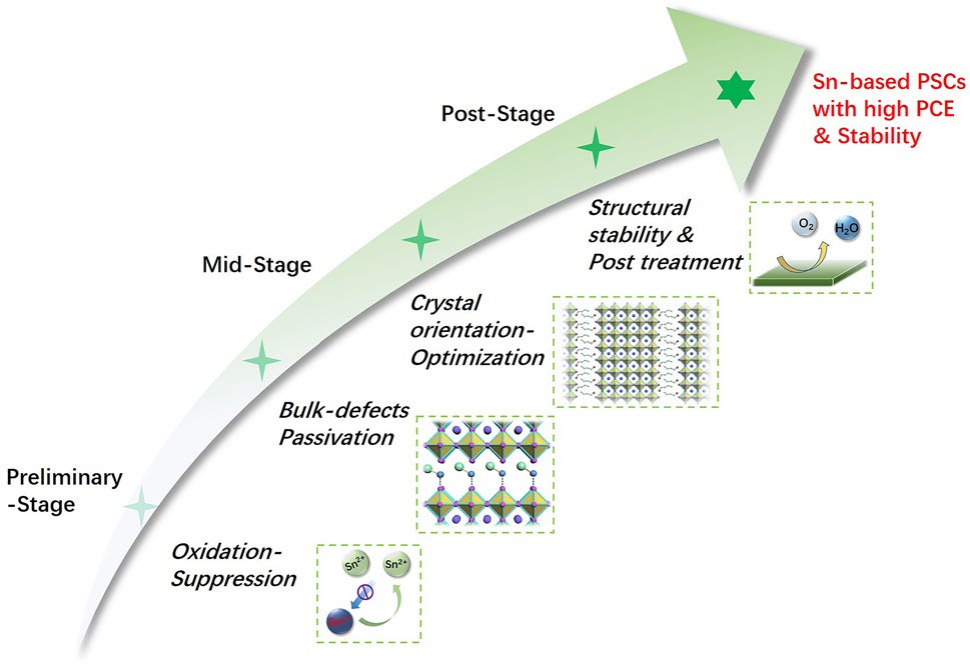
Schematic illustration of ligands acting at different stages of the thin film fabrication process by different mechanisms

In this review, a systematic presentation of ligand engineering in Sn-based perovskites and solar cells is dedicated. Ligands act at different stages of the thin film fabrication process by different mechanisms, which can be divided into a preliminary stage (precursors), a mid-stage (film preparation), and a post-stage (film stability), as schematically illustrated in Fig. 1. (i) The ligand possesses antioxidant effect in the precursor solutions, which aims to reduce the oxidation of Sn^2+^ in the source phase. (ii) The ligand assists film formation during the preparation process, which depends on the coordination effect between the ligand and the perovskite, and the relevant strategies in the mid-stage. (iii) The ligand induces dimensional engineering as film growing completes, where the heterojunction structures and 2D and quasi-2D structures are highlighted. (iv) The ligand favors improving the stability of Sn-based PSCs, which must be solved in the face of commercialization. At last, we provide our insights and prospects toward further performance optimization in view of ligand strategy, aiming at making advances in the field of environmentally friendly perovskite solar cells.

## Ligands for Antioxidation at Preliminary Stage

The suppression of Sn^2+^ oxidation at the source stage during the fabrication process is considered the key point for improving the photovoltaic performance of Sn-based PSCs. The most commonly utilized antioxidant ligand is SnX_2_ (X = F, Cl, Br, I). In 2012, Kanatzidis et al*.* proceed a pioneering work to use CsSnI_3_ as an efficient hole-transporting material in solid-state dye-sensitized solar cells, and in the same work, SnF_2_ was doped into CsSnI_3_ to increase the efficiency of relevant cells [22]. Soon after, Mathews et al*.* first used SnF_2_ in CsSnI_3_ PSCs to reduce the intrinsic Sn vacancies [23]. Since then, the effectiveness of SnX_2_ additive has been proven over time by abundant relevant works. Simultaneously, ligands for coordination with SnX_2_ additives have been widely studied. Furthermore, hydrazine and its derivatives are widely known as strong reducing agents and also strong base which could potentially prevent or suppress the oxidation of Sn^2+^ at the source stage, which should also be noticed for further investigation.

### SnX_2_ Engineering

In recent years, most of the reported works have employed SnX_2_ as a standard process. After the first application of SnF_2_ in CsSnI_3_, Mathews et al*.* tried to utilize FASnI_3_ with low bandgap (1.41 eV) as a light-absorbing layer. The addition of SnF_2_ in FASnI_3_ improved the coverage of perovskite layer over the mesoporous TiO_2_ layer and thus resulted in a significant increase of photocurrent density. However, with the increase of SnF_2_ concentration, nano-platelet-like structures could be observed on the film surface, which might on the contrary cause the reduced performance of the resulting PSCs [24]. To study the properties of CsSnI_3_ perovskite upon the addition of SnF_2_, Falaras et al*.* carried out powder X-ray diffraction. Due to the irreversible oxidation caused by the exposure to air, CsSnI_3_ perovskite underwent rapid phase transformation from the black orthorhombic phase (B-$\gamma$-CsSnI_3_) to the yellow orthorhombic phase (Y-CsSnI_3_). The phase transition rate could be significantly lower in the SnF_2_-containing material, comparing with the pure perovskites [25]. Besides CsSnI_3_, Kanatzidis et al*.* found that with the addition of SnF_2_ into precursor solution, the fluorescence lifetime and carrier diffusion lengths of MASnI_3_ films were enhanced, which indicated the reduced defect concentration [26]. Compared with MASnI_3_ film prepared with SnF_2_, the pristine film without SnF_2_ had a significantly blue-shifted absorption edge, which is due to the Burstein–Moss shift induced by a significant unintentional hole doping [27]. According to the summary of early work, SnF_2_ is believed to affect many different properties of the Sn-based perovskites, including film morphology, doping, inhibiting the formation of unwanted phases, stability of materials, and energy-level matching [28].

Other than SnF_2_, Hatton et al*.* reported that the use of excess SnI_2_ could be an effective strategy for improving both the stability and efficiency of PSCs simultaneously, which resulted from a Sn-rich environment during CsSnI_3_ preparation [29]. After that, they managed to compare the stability of CsSnI_3_ perovskite with SnF_2_, SnCl_2_, SnBr_2_, and SnI_2_ as additives, respectively. The result indicated that SnCl_2_ benefited the highest stability of resulted films. High-resolution X-ray photoelectron spectroscopy (HRXPS) analysis revealed that there was only one Cl 2*p* environment in CsSnI_3_ film with SnCl_2_ additive as these peaks have the identical binding energy as SnCl_2_, which is consistent with the conclusion that Cl is not incorporated into the perovskite structure. This suggests that SnCl_2_ is presented as a layer of films or particles on the surface of the perovskite crystal. Based on these results, they built the hole-transport-layer-free PSCs with the structure of ITO/CsSnI_3_/PC_61_BM/BCP/Al, and PSCs containing SnCl_2_ gained the highest PCE. The increment may be due to a layer of SnCl_2_ buried at the ITO/CsSnI_3_ interface to perturb the interfacial energetics by modifying the surface potential at the ITO electrode [30]. In 2020, Han et al*.* found that the introduction of excess SnF_2_ and SnCl_2_ simultaneously would form an amorphous-polycrystalline structure composed of a Sn triple-halide (Sn-3X, F^−^, Cl^−^, and I^−^) amorphous layer and CsFASnI_3_ polycrystals. A well-crystallized Sn-3X film covered by the amorphous layer of 3–4 nm in thickness could be observed under transmission electron microscopy (TEM). Such structure acted as a blocking layer of moisture, oxygen, and ion diffusion. As a result, the corresponding Sn-based PSCs exhibited a PCE of 10.4%, with a *V*_*oc*_ of 0.64 V, a *J*_*sc*_ of 21.6 mA cm^−2^ and an FF of 75.2% (certificated as 10.08%), along with the outstanding stability of over 1000 h kept in N_2_ environment [31]. After that, they also utilized tin(II) acetate (Sn(Ac)_2_) to replace conventional SnF_2_ additive in precursor. They demonstrated that tin(II) acetate (Sn(Ac)_2_) not only owned all the benefits of SnF_2_, but also markedly improved the stability and charge extraction of tin-based PSCs. The carboxyl group of Sn(Ac)_2_ could coordinate effectively with Sn cations, leading to the passivation of the un-coordinate Sn as well as creating a weakly polarized protective layer that reduced extrinsic degradation. The FASnI_3_–Sn(Ac)_2_ film exhibited an increase in PL intensity and a twice longer carrier lifetime (9.8 ns) than the control one (4.1 ns). Finally, the FASnI_3_–Sn(Ac)_2_ devices yielded a PCE of 9.93% and an efficiency loss of less than 10% after 1000 h operation at the maximum power point [32].

More recently, Abate et al*.* studied the fluoride chemistry in Sn-based perovskites. Using nuclear magnetic resonance (NMR), they interestingly uncovered that SnF_2_ and Sn^4+^ in precursor undergo a simple ligand exchange reaction to produce colorless SnF_4_ in solution, other than a redox reaction. Fluoride is a small, non-polarizable, and electronegative anion that exhibits a more intense affinity for a cation with a similar nature, namely Sn^4+^, and Sn^4+^, is smaller and more electronegative than its reduced analogue Sn^2+^. With this affinity, fluorides could complex Sn^4+^ as soon as it is generated, whether from O_2_ or DMSO-driven oxidation. Furthermore, the selective complexation of fluoride and Sn^4+^ could hinder the ability of forming any perovskite-like complex in solution. As a result, the point defects in the perovskite lattice caused by Sn^4+^ are significantly reduced. Other SnX_2_ (X = Cl, Br, I) were also studied; the conclusion came that SnCl_2_ had the same effect as SnF_2_, leading to selective complexation with Sn^4+^ and forming SnCl_4_. These results illustrated that hard Lewis base (chloride and fluoride) could prevent the formation of Sn^4+^ in precursor solution and introduction into the Sn-based perovskite film by two mechanisms: complexation with Sn^4+^ and antioxidation property [33]. After that, Powalla et al*.* investigated the spatial distribution of SnF_2_ additive within FASnI_3_ films deposited on top of PEDOT:PSS hole-transport layer (HTL). The results of time-of-flight secondary ion mass spectrometry (ToF–SIMS) measurements on SnF_2_-modified FASnI_3_ films revealed that fluoride mainly accumulated at the perovskite surfaces, and especially at the PEDOT:PSS/perovskite interface. XPS/HAXPES spectra indicated the existence of SnS_x_ interlayer at PEDOT:PSS/perovskite interface with the thickness of 1.2 nm, which was induced by a chemical reaction with sulfur-containing groups at the PEDOT:PSS surface [34].

### Ligands for SnX_2_ Additive

Despite the improved performance of film and devices, excess SnX_2_ is suspected to induce phase separation and micro-sized aggregates. To prevent such phenomenon, ligands aiming at coordinating with SnX_2_ need to be considered. Table 1 summarizes the reported ligands in recent years.

**Table 1:** Chemical structure of ligands for SnX_2_ and PV parameters of corresponding PSCs

Perovskite	Ligand	*J*_*sc*_ [mA cm^−2^]	*V*_*oc*_ [V]	FF [%]	PCE [%]	Stability	Refs.
FASnI_3_	Pyrazine	23.7	0.32	63	4.8	25% RH, encapsulated, shelf life, 100 days (98%)	[35]
CsSnIBr_2_	Hypophosphorous acid	17.4	0.31	57	3.2	20% RH, encapsulated, shelf life, 77 days (103%)	[36]
FASnI_3_	Trimethylamine	22.45	0.47	67.8	7.09	N_2_, shelf life, 20 days (80%)	[37]
FASnI_3_	Potassium salt of hydroquinone sulfonic acid	17.64	0.552	69.4	6.76	20% RH, unencapsulated, shelf life, 500 h (80%)	[42]
FASnI_3_	Gallic acid	19.75	0.64	71.4	9.03	20% RH, unencapsulated, shelf life, 1000 h (80%)	[43]
FA_0.75_MA_0.25_SnI_3_	1,4-bis(trimethylsilyl)-2,3,5,6-tetramethyl-1,4-dihydropyrazine	22.0	0.76	69	11.5	N_2_, unencapsulated, shelf life, 50 days (100%)	[41]
FASnI_3_	Anilinium hypophosphite	22.25	0.37	66.36	5.48	–	[38]
FA_0.5_MA_0.45_PEA_0.05_SnI_3_	Anilinium hypophosphite	25.21	0.48	57.16	6.87	N_2_, shelf life, 30 days (97%)	[39]
CsSnI_3_	2-aminopyrazine	21.7	0.40	59	5.12	N_2_, unencapsulated, shelf life, 60 days (92%)	[44]

*RH* relative humidity; *MPPT* max power point track

The addition of pyrazine provides a binding affinity to SnF_2_ and is also easily removed during annealing due to its low boiling point of 115 °C. Seok et al*.* found that pyrazine limited the phase separation caused by SnF_2_, which effectively reduced the Sn vacancies. The complexation of pyrazine and SnF_2_ promoted the homogenous dispersion of SnF_2_ into perovskite [35]. Wang et al*.* fabricated all-inorganic CsSnIBr_2_ perovskite film and HTL-free PSCs. The incorporation of hypophosphorous acid (HPA) strongly coordinated with Sn^2+^ through the P–O bond, promoted the migration of SnF_2_, eliminated the residual SnF_2_ in the grain boundary, which resulted in a pure phase CsSnIBr_2_ perovskite [36]. Jen et al*.* attempted to realize FASnI_3_ perovskite via a sequential deposition route. In the first step deposition, additional Lewis base trimethylamine (TMA) was employed to form SnY_2_–TMA complexes (Y = I^−^, F^−^), followed by the deposition of FAI. Such intermediate complexes could help facilitate the formation of homogeneous film. On the other hand, SnY_2_–TMA complexes had relatively larger size and weaker affinity with SnI_2_ than FA^+^ and therefore formed dense and compact FASnI_3_ film with crystalline domain larger than 1 $\mathrm{\mu m}$ [37]. Wang et al*.* reported the incorporation of anilinium hypophosphite (AHP) into FASnI_3_ and FA_0.5_MA_0.45_PEA_0.05_SnI_3_, respectively. The interaction between AHP and SnF_2_ resulted in the formation of a double-salt complex (Sn(H_2_PO_2_)_2_$\cdot$SnF_2_), which was proved to eliminate the phase separation caused by SnF_2_ in the perovskite and passivate the perovskite films [38, 39]. An amine complex, CH_3_NH_3_I·3CH_3_NH_2_ (MAI·3MA), was introduced simultaneously with SnF_2_ to hinder the major issue caused by the oxidation of Sn^2+^ to Sn^4+^. Like the aforementioned TMA additive, the presence of electron-donating additive MAI·3MA would tend to favor the formation of SnI_2_ complexes and thus slow down the consumption as a product in the global crystallization reaction. The resulted optimized films were more stable with decreased defect density (from 6.50 $\times$ 10^16^ cm^−3^ for pristine films to 2.63 $\times$ 10^16^ cm^−3^ for target films). Meanwhile, they fabricated PSCs with an inverted structure and gained a PCE of 9.53%. The encapsulated devices showed an impressive stability under continuous light soaking in ambient air condition for 1000 h [40].

Later, Wakayama et al*.* fabricated Sn(IV)-free perovskite films with strong photoluminescence and prolonged decay lifetimes by in situ Sn(0) nanoparticle treatment. It was found that the introduction of 1,4-bis(trimethylsilyl)-2,3,5,6-tetramethyl-1,4-dihydropyrazine (TM-DHP) in precursor solution would selectively react with SnF_2_ over SnX_2_ (X = I, Br, Cl) to form Sn(0) nanoparticles, which was believed to result from the strong affinity between the trimethylsilyl groups and the fluoride. Combined with the interface modification by EDA and PCBM, the corresponding PSCs showed a PCE up to 11.5% [41].

**Fig. 2:**
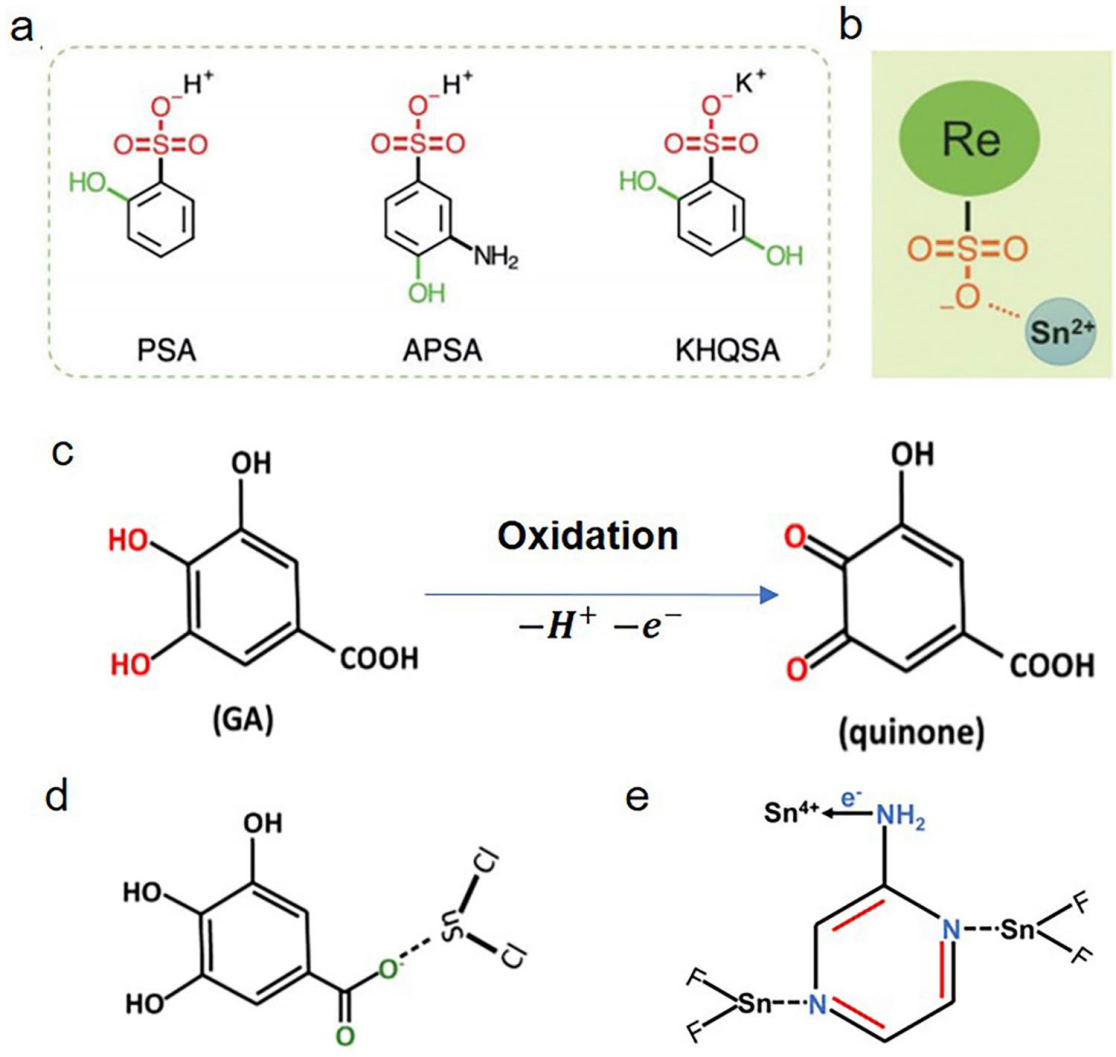
**a** Molecular structures of PSA, APSA, and KHQSA. **b** Schematic illustration of the interaction between the additive molecule and Sn^2+^ ion. Reproduced with permission from Ref. [42]. **c** Chemical reaction showing the oxidation of GA to quinone when exposed to air. **d** Schematic illustration of the interaction between GA and SnCl_2_. Reproduced with permission from Ref. [43]. **e** Schematic illustration of the interaction between 2-aminopyrazine (APZ) and SnF_2_. Reproduced with permission from Ref. [44]

In the study of SnCl_2_ additives, Yan et al*.* reported the introduction of hydroxybenzene sulfonic acid or its salt along with SnCl_2_ additive. From a variety of options, Yan and co-workers chose the potassium salt of hydroquinone sulfonic acid (KHQSA, corresponding chemical structure in Fig. 2a), in which the two hydroxyl groups (–OH) have high antioxidant activity and the sulfonate group (–SO_3_^−^) could interact with Sn^2+^ via coordination interactions and electrostatic attraction (Fig. 2b). The coordination of sulfonate group and Sn^2+^ ion enabled the in situ encapsulation of the FASnI_3_ grains with a SnCl_2_-additive antioxidant outer layer, rendering a significantly improved oxidation stability of the FASnI_3_ film and the corresponding PSCs [42]. Later, the same group put their attention on introducing gallic acid (GA). As previous report, the hydroxyl groups (–OH) attached to the aromatic ring endow the antioxidant property of GA. As shown in Fig. 2c, –OH can protect Sn-based perovskite by effectively scavenging oxygen through the donation of hydrogen atoms and electrons. Due to the Lewis acidity of SnCl_2_, it readily accepts lone pairs (e.g., O atoms from GA) and coordinates with GA (as shown in Fig. 2d). The formation of SnCl_2_–GA complex could envelop the perovskite grain surface and restrict excess SnCl_2_ aggregation. Furthermore, SnCl_2_ and its complexes (KHQSA and GA) have a much larger band gap than that of FASnI_3_ film, which prohibits the transfer of both holes and electrons to the electron transport layer PCBM. Considering the band structure of complexes, the tunneling current density, *J*, can be calculated by the following equation:

1 $$ J \sim E^{2} \exp \left[ { - \frac{{4\sqrt {2m^{*} } \left( {q\phi_{B} } \right)^{\frac{3}{2}} }}{3q\hbar E}} \right] $$

where *E* is the electric field, ${m}^{*}$ is the effective mass, and ${\phi }_{B}$ is the barrier height. It could be observed that a lower barrier height would lead to a higher tunneling current density. Thus, the SnCl_2_–GA complex with lower conduction band minimum (CBM) showed much better performance of PSCs [43].

Recently, inorganic CsSnI_3_ have received more attention due to its small optical bandgap. Wang et al*.* studied the coadditive 2-aminopyrazine (APZ) to form SnF_2_–APZ complex in CsSnI_3_ precursor, aiming at restrain Sn^2+^ oxidation and improve device performance. As shown in Fig. 2e, SnF_2_ have a strong binging affinity with APZ to form complexes. Such coordination was proved by Fourier-transform infrared (FTIR) spectroscopy; the characteristic C=N stretching vibration shifted to a higher wavenumber than that without SnF_2_. The shift could be attributed to the $d\to {\pi }^{*}$ back donation from SnF_2_ to APZ ring. The ligand coadditive engineering strategy enabled a homogeneous distribution of SnF_2_, which avoided the aggregation at grain boundaries [44].

### Ligands with Reducing Capability

#### Hydrazine and Its Derivatives

**Fig. 3:**
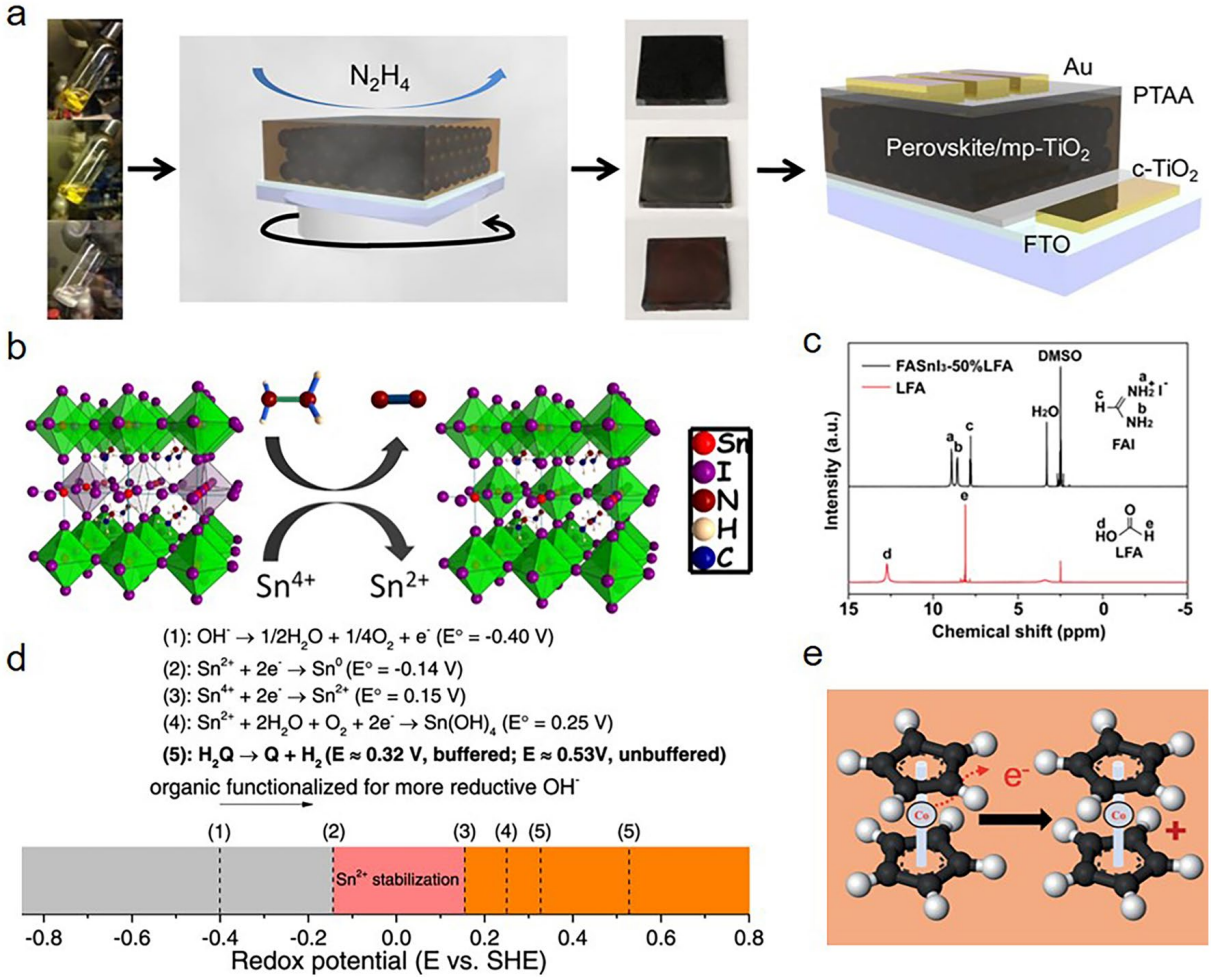
**a** Scheme of reducing vapor atmosphere process of device fabrication. **b** Proposed possible mechanism of hydrazine vapor reaction with Sn-based perovskite materials. Reduction process: $2{\mathrm{SnI}}_{6}^{2-}+{\mathrm{N}}_{2}{\mathrm{H}}_{4}\to 2{\mathrm{SnI}}_{4}^{2-}+{\mathrm{N}}_{2}+4\mathrm{HI}$. Reproduced with permission from Ref. [45]. **c** Proton nuclear magnetic resonance spectra of the FASnI_3_-50%LFA perovskite film dissolved in deuterated DMSO solution. Reproduced with permission from Ref. [52]. **d** Scheme of redox reactions related to Sn chemical species and H_2_Q. Reproduced with permission from Ref. [56]. **e** Schematic illustration of CoCp_2_’s redox property. CoCp_2_ has 19 valence electrons and it tends to lose this “extra” electron to yield an 18-electron cation known as CoCp_2_^+^. Reproduced with permission from Ref. [58]

Kanatzidis et al*.* firstly used hydrazine vapor (N_2_H_4_) treatment to suppress the high-oxidation Sn^4+^ formation during the preparation of Sn perovskite solar cells (MASnI_3_, CsSnI_3_, and CsSnBr_3_ as the representative absorbers) (Fig. 3a). Instead of introducing hydrazine directly into the perovskite solutions, a hydrazine vapor atmosphere afforded a favorable proper reduction of Sn^4+^ via the reduction process as $2{\mathrm{SnI}}_{6}^{2-}+{\mathrm{N}}_{2}{\mathrm{H}}_{4}\to 2{\mathrm{SnI}}_{4}^{2-}+{\mathrm{N}}_{2}+4\mathrm{HI}$, as shown in Fig. 3b. The hydrazine vapor not only avoided the overreduction of Sn^2+^ to metallic Sn, but also reduced the Sn^4+^ impurities and suppressed the unfavorable oxidation of Sn^2+^. The reduction of Sn^4+^ to Sn^2+^ decreased the amount of Sn^2+^ vacancies (V_Sn_), thus lowering the undesirable p-type conductivity of tin perovskite films. The XPS measurement further confirmed that by using the reducing hydrazine vapor, the Sn^4+^/Sn^2+^ ratios decreased by 45.8%, 21.5%, and 20.8% in MASnI_3_, CsSnI_3_, and CsSnBr_3_ films, respectively. More importantly, almost no Sn^0^ was observed after the etching process [45]. Similarly, they combined the excess SnI_2_ with hydrazine vapor treatment to effectively reduce the p-type conductivity and significantly improved the solar cell performances of all the ASnI_3_ materials. The optimized CsSnI_3_ device with a PCE of 4.81% was the highest one among all-inorganic Pb-free perovskite solar cells at that time [46].

Inspired by these studies, more hydrazine derivatives have been applied to Sn-based perovskites. Islam et al*.* enabled a reduced concentration of Sn^4+^ content by 20% via the introduction of hydrazinium chloride (N_2_H_5_Cl) in precursors, leading to a pinhole-free uniform film. The N_2_H_5_Cl-treated PSCs boosted the PCE up to 5.4% with *V*_*oc*_ of 0.455 V and FF of 0.67 [47]. Likewise, Hou et al*.* doped hydrazine monohydrobromide (N_2_H_5_Br) into FASnI_3_-based perovskite precursor solutions as a reducing agent to reduce the defects and trap states in as-formed perovskites, as well as inhibit the formation of Sn^4+^, and increase the open circuit voltage by widening the bandgap of perovskite. As a consequence, an excellent PCE of 7.81% was achieved for the optimized device, which represented a relative 39.5% improvement compared to the best reference one [48].

Jiang et al*.* reported the use of dihydrotriazine ((N_2_H_4_)_3_(HI)_2_, THDH) as an additive to fabricate high-efficiency FASnI_3_-based solar cells. The hydrazine released from THDH in solution was effective in reducing the Sn^4+^ content from 35.9 to 9.1%, as measured by XPS spectra, and hydrazinium iodide (N_2_H_5_I) left from THDH remained in the resulted FASnI_3_ films would act as a stabilizer against oxidation. As a consequence, the 3% THDH-treated FASnI_3_ exhibited a maximum PCE of 8.48% with good reproducibility [49].

Huang et al*.* introduced phenylhydrazine hydrochloride (PHCl) into FASnI_3_ perovskite films to reduce the amount of Sn^4+^ and improve stability. The reductive PH^+^ could successfully incorporate into the crystal lattice and lead to lattice expansion without forming a 2D structure. The FASnI_3_-5.0% PHCl film showed 3 times longer carrier lifetime and a built-in voltage ≈ 3.5 times higher than the control film without PHCl. Besides, combined with the hydrophobic phenyl group of PHCl, the unencapsulated device maintained its initial efficiency for over 110 days in a glove box [50].

Recently, Liu et al*.* revealed that the reducing phenylhydrazine cation (PhNHNH_3_^+^) and halogen anions (Cl^–^ and Br^–^) could successfully improve the illumination stability of FASnI_3_ perovskite solar cells. The introduction of PhNHNH_3_^+^ could effectively improve the chemical potential of the film, which inhibited the oxidation of Sn^2+^. Moreover, the introduction of a tiny amount of Cl^–^ was necessary to improve film morphology and the doping of Br^–^ further optimized the device performance. Hence, the PHCl–Br-based FASnI_3_ device achieved a record PCE of 13.4% (certified 12.4%) with a remarkably improved *V*_*oc*_ of 0.81 V and superior long-term device durability [51].

#### Other Reducing Agents

Besides hydrazine and its derivatives, Han et al*.* first introduced the volatile liquid formic acid (LFA) into FASnI_3_ perovskite precursor solution to suppress oxidation of Sn^2+^ to Sn^4+^. The incorporation of LFA significantly reduced the Sn^4+^ content from 23.7 to 14% in the FASnI_3_ perovskite films. Meanwhile, the stronger PL intensity and longer carrier lifetime (12.4 ns) indicated that the background doping and trap density were efficiently alleviated. More importantly, by confirming the ^1^H NMR spectra in Fig. 3c, LFA was absent in the final FASnI_3_–LFA perovskite films, indicating that LFA did not sacrifice the crystallinity or remain in the FASnI_3_ perovskite films. Finally, the FASnI_3_ PSCs devices fabricated with LFA delivered an efficiency of over 10% with improved reproducibility [52]. Recently, Liao et al*.* employed two different aromatic carboxylic acid molecules, 4,4′-biphenyldicarboxylic acid (BP2Ac) and biphenyl-3,3′,5,5′-tetracarboxylic acid (BP4Ac), into the perovskite precursor solution. By adjusting pH of the precursor solution, the oxidation of Sn^2+^ could be suppressed. The fact that the free energy of the redox reaction ($\Delta$*G*^0^) is negative and the reaction can proceed spontaneously in an alkaline environment showed that Sn^2+^ is unstable there. On the other hand, when $\Delta$*G*^0^ is positive in an acidic environment and the redox reaction cannot proceed spontaneously, the oxidation of Sn^2+^ could be inhibited. The content of Sn^4+^ from XPS measurement was effectively suppressed when the acidity of the precursor solution increased, and the highest PCE was obtained when pH = 4.62. However, it should be noticed that when pH further lessened, PCE declined, which was mainly attributed to the high doping concentration and the detrimental effect of acidity on perovskites [53].

Yan et al*.* introduced ammonium hypophosphite (AHP, NH_4_H_2_PO_2_) additive to treat the FASnI_3_ perovskite precursor to suppress the oxidation of Sn^2+^. They found that AHP can prohibit the oxidation of Sn^2+^ in perovskites through the following reaction:

2 $${\mathrm{Sn}}^{4+}+3{\mathrm{H}}_{2}{\mathrm{PO}}_{2}^{-}\to {\mathrm{Sn}}^{2+}+2{\mathrm{HPO}}_{3}^{2-}+{\mathrm{PH}}_{4}^{+}$$

Meanwhile, the addition of AHP would inhibit the needle-like aggregates formed on the film due to phase separation of SnCl_2_. Moreover, they used CuSCN as an inorganic hole transporting material to form a good energy-level alignment with the FASnI_3_ PSCs. Consequently, the devices with 5% AHP showed a PCE of 7.34% with pronounced enhancement of the long-term stability [54].

In 2020, Diau et al*.* fabricated a hole-transporting materials (HTM)-free carbon structure Sn-PSCs with uric acid (UA) as a natural antioxidant additive. It was found that 10% UA could effectively reduce the Sn^2+^ oxidation and decrease the carrier recombination, suggesting an ideal strategy to applicate the inexpensive and available antioxidants that have certain functional groups like OH^−^, NH_2_, or SO_3_^−^ [55].

Later, Xu et al*.* reported that hydroquinone (H_2_Q), a chemically reductive organic molecule, exhibit the ability to alleviate the oxidation of Sn^2+^ and retard the degradation of MASnI_3_ devices in a dry air environment. From the electrochemistry perspective, the oxidation of H_2_Q was more spontaneous than Sn^2+^, in other words, H_2_Q was an effective reducing agent for preserving Sn^2+^ in MASnI_3_. The fact that the reducing effects of H_2_Q can be rationalized that *sp*^2^-hybridized C atom and OH share electrons due to the covalent bond, thus resulting the bonded electrons easier to lose than individual OH^−^ ions. XPS measurement (the content of Sn^4+^: 8.25% for pristine MASnI_3_, 5.36% for H_2_Q:MASnI_3_) further confirmed that –OH is oxidized through dehydrogenation reaction to become ketone (C=O), thereby sacrificially suppressing the oxidation of Sn^2+^ to Sn^4+^. From the electrochemistry perspective, the working mechanism of H_2_Q suppressing the Sn^2+^ oxidation could be revealed by comparing the redox potentials of Sn species and H_2_Q. As illustrated in Fig. 3d, the oxidation of Sn^2+^ with both water and oxygen possessed a 0.25 V potential (referenced to a standard hydrogen electrode (SHE), reaction (4)). Such reaction potential was obviously smaller than the oxidation potential of H_2_Q (0.32 V vs SHE at 25 °C buffered condition, and 0.53 V vs SHE under unbuffered condition). Therefore, the oxidation of H_2_Q was more spontaneous than Sn^2+^, making H_2_Q an effective reducing agent in suppressing Sn^2+^ oxidation [56].

Huang et al*.* reported a purification method that Sn powder could purify SnI_2_ with 99% purity via the simply redox reaction: ${\mathrm{Sn}}^{4+}+\mathrm{Sn}\to 2{\mathrm{Sn}}^{2+}$. Consequently, the optimized device achieved a PCE of 6.75%, with a *V*_*oc*_ of 0.58 V, a *J*_*sc*_ of 17.5 mA cm^−2^, and an FF of 66.3%, which was even higher than the device fabricated from SnI_2_ with a high purity of 99.999%. This work highlights the importance of the purity of SnI_2_, especially the Sn^4+^ impurity to the reproducibility and validation of Sn-based PSCs [57].

Wang et al*.* introduced cobaltocene (CoCp_2_) as a chemical doping agent that could donate the electron to CsSnI_3_ to offer the capability of suppressing Sn^2+^ oxidization and lower the trap density. As shown in Fig. 3e, CoCp_2_ is a commonly one-electron reducing agent which can easily give up an extra electron from the metal cobalt to form an 18-electron cation with high stability. The XPS measurement confirmed the occurrence of a substantial electron transfer from CoCp_2_ to CsSnI_3_ and thus reducing the self-doping effect. In particular, the average lifetime was enhanced from 3.66 to 8.71 ns and the trap density decreased from 1.08 $\times$ 10^19^ to 4.48 $\times$ 10^18^ cm^−3^ after incorporating CoCp_2_ [58].

## Effect of Ligands on Film Fabrication

Sn-based perovskite films possess intrinsic high defects mainly due to Sn vacancies and p-type doping, aroused from the facile oxidation of the metastable Sn^2+^ and ultrafast crystallization behavior, which heavily influences carrier transport through the formation of non-radiative recombination centers. Various ligands that successfully suppressed the bulk defects in Sn-based perovskite films during the intermediate state (film preparation) have been widely reported. With different functional groups, incorporated ligands tend to coordinate with either Sn cations to suppress the formation of Sn vacancies, or halide anions to anchor the perovskite lattice, or alternatively, with both of them simultaneously. In this section, we provide a review on ligands passivating bulk defects based on the coordination objective ions. The representative reports are listed in Table 2.

**Table 2:** Ligands passivating bulk defects based on the coordination objective ions and PV parameters of corresponding PSCs

Perovskite	Ligand	*J*_*sc*_ [mA cm^−2^]	*V*_*oc*_ [V]	FF [%]	PCE [%]	Stability	Refs.
FASnI_3_	2-fluoro-phenethylammonium iodide	21.53	0.69	68.46	10.17	N_2_, unencapsulated, continuous 1 sun irradiation, 1600 h (85%)	[64]
FASnI_3_	Ethylenediammonium diiodide	21.30	0.583	71.8	8.9	N_2_, encapsulated, shelf life, 2000 h, slight degradation	[59]
FASnI_3_	Butylammonium iodide	18.00	0.44	69.4	5.5	N_2_, encapsulated, shelf life, 2000 h (90%)	[59]
FASnI_3_	Ethane-1,2-diamine	22.80	0.56	74	9.37	N_2_, shelf life, 7 days (108%)	[60]
FASnI_3_	PTN-Br	20.66	0.57	67.40	7.94	N_2_, encapsulated, continuous UV light irradiation, 5 h (66%)	[70]
FASnI_3_	Pentafluorophen-oxyethylammonium iodide	21.59	0.667	75.1	10.81	In air, light soaking of AM 1.5G, 500 h, maintained its original efficiency	[66]
FASnI_3_	2-phenoxyethylamine bromide	22.44	0.86	74.20	14.32	N2, unencapsulated, MPPT, 600 h (no obvious degradation)	[67]
FASnI_3_	3-phenyl-2-propen-1-amine	23.34	0.56	73.5	9.61	N_2_, unencapsulated, shelf life, 1440 h (92%)	[63]
FASnI_3_	n-propylammonium iodide	22.37	0.73	72.0	11.78	N_2_, encapsulated, MPPT, 1000 h (95%)	[61]
FA_0.98_EDA_0.01_SnI_3_	Ethylammonium halides	21.46	0.79	73.73	12.50	N2, unencapsulated, MPPT, 100 h (no obvious degradation)	[62]
FASnI_3_	Trifluoroethylamine iodide	22.11	0.617	68.47	9.34	N_2_, unencapsulated, MPPT, 500 h (90%)	[65]
CsSnI_3_	Cobaltocene	18.24	0.36	0.46	3.0	N_2_, shelf life, 100 h, no obvious degradation	[58]
FASnI_3_	8-hydroxyquinoline	22.24	0.493	65.19	7.15	N_2_, unencapsulated, shelf life, 800 h (90%)	[89]
FA_0.75_MA_0.25_Sn(I_0.75_Br_0.25_)_3_	Melamine	21.17	0.69	70.36	10.30	N_2_, unencapsulated, shelf life, 1300 h (85%);In air, unencapsulated, shelf life, 30 h (78%)	[90]
FASnI_3_	Poly (ethylene-co-vinyl acetate)	22.80	0.523	64.69	7.72	60% RH, unencapsulated, shelf life, 48 h (62.4%)	[76]
FASnI_3_	Trifluoroacetamide	22.24	0.687	76.84	11.74	N_2_, unencapsulated, shelf life, 1800 h (86%)	[79]
FASnI_3_	IO-4Cl	22.26	0.68	75.91	11.49	N_2_, unencapsulated, shelf life, 2500 h (90%)	[80]
FASnI_3_	4-fluorobenzohydrazide	21.10	0.598	75.10	9.47	In air, encapsulated, MPPT, 600 h (93%)	[81]
FASnI_3_	Formamidine acetate	23.20	0.59	72.76	9.96	N_2_, unencapsulated, continuous illumination, 1600 h (82%)	[93]
FA_0.75_MA_0.25_Sn(I_0.75_Br_0.25_)_3_	Formamidine acetate	21.72	0.797	71.84	12.43	N_2_, shelf life, 2000 h (94%)	[94]
FASnI_3_	n-butylammonium acetate	22.20	0.65	71.6	10.40	N_2_, shelf life, 1000 h (96%)	[95]
FASnI_3_	Fluorinated-perylene diimide	20.81	0.65	69.62	9.49	N_2_, continuous light soaking, 2880 h (80%)	[83]
FASnI_3_	2-cyano-3-[5-[4-(diphenylamino) phenyl]-2-thienyl]-propenoic acid	21.60	0.63	74.70	10.17	30% RH, encapsulated, MPPT, 1000 h (90%)	[82]
FASnI_3_	2-cyano-3-[5-(2,4-difluorophenyl)-2-thienyl]- propenoic acid	20.45	0.57	69.10	8.05	–	[82]
FASnI_3_	2-cyano-3-[5-(2,4-dimethoxyphenyl)-2-thienyl]- propenoic acid	21.05	0.59	72.10	8.96	–	[82]
FASnI_3_	5-ammonium valeric acid	18.89	0.592	62.30	7	50% RH, encapsulated, MPPT, 100 h (100%)	[73]
FASnI_3_	Poly(vinyl alcohol)	20.371	0.632	69.3	8.92	In air, encapsulated, MPPT, 400 h (100%)	[75]
FASnI_3_	Hexamethylenediamine diiodide	21.46	0.514	68.87	7.6	N_2_, unencapsulated, shelf life, 550 h (80%)	[68]
PEA_0.1_FA_0.9_SnI_3_	Aminoguanidine hydrochloride	19.65	0.56	68.94	7.3	N_2_, unencapsulated, shelf life, 30 days (90%)	[69]
FASnI_3_	Piperazine dihydriodide	21.85	0.69	75.1	11.39	In air, MPPT, 500 h (90%)	[72]
EA_0.1_FA_0.9_SnI_3_	1-butyl-3-methylimidazolium bromide	19.86	0.70	72.36	10.09	N_2_, unencapsulated, shelf life, 1200 h (85%)	[96]
FASnI_3_	Fulleropyrrolidine with triethylene glycol monoethyl ether side chain	20.70	0.625	67.8	8.78	In air, encapsulated, continuous illumination, 1000 h (65%)	[86]
FASnI_3_	Graphite phase-C_3_N_4_	20.68	0.621	66.68	8.56	N_2_, shelf life, 1000 h (91%)	[87]
CsSnI_3_	Thiosemicarbazide	19.7	0.63	66.1	8.2	N_2_, encapsulated, continuous illumination, 500 h (90%)	[77]
Cs_0.1_FA_0.9_SnI_3_	2-thiophenemethylammonium iodine	24.12	0.521	72.02	9.06	N_2_, encapsulated, shelf life, 35 days (90%)	[88]
PEA_0.15_FA_0.85_SnI_3_	Chlorofullerene, C_60_Cl_6_	20.31	0.86	76.0	13.30	In air, unencapsulated, 10 h (95%)	[97]

*RH* relative humidity; *MPPT* max power point track

### Ligands for Coordination with Halide Anions

#### Coordination by Ligands with Amino Group

**Fig. 4:**
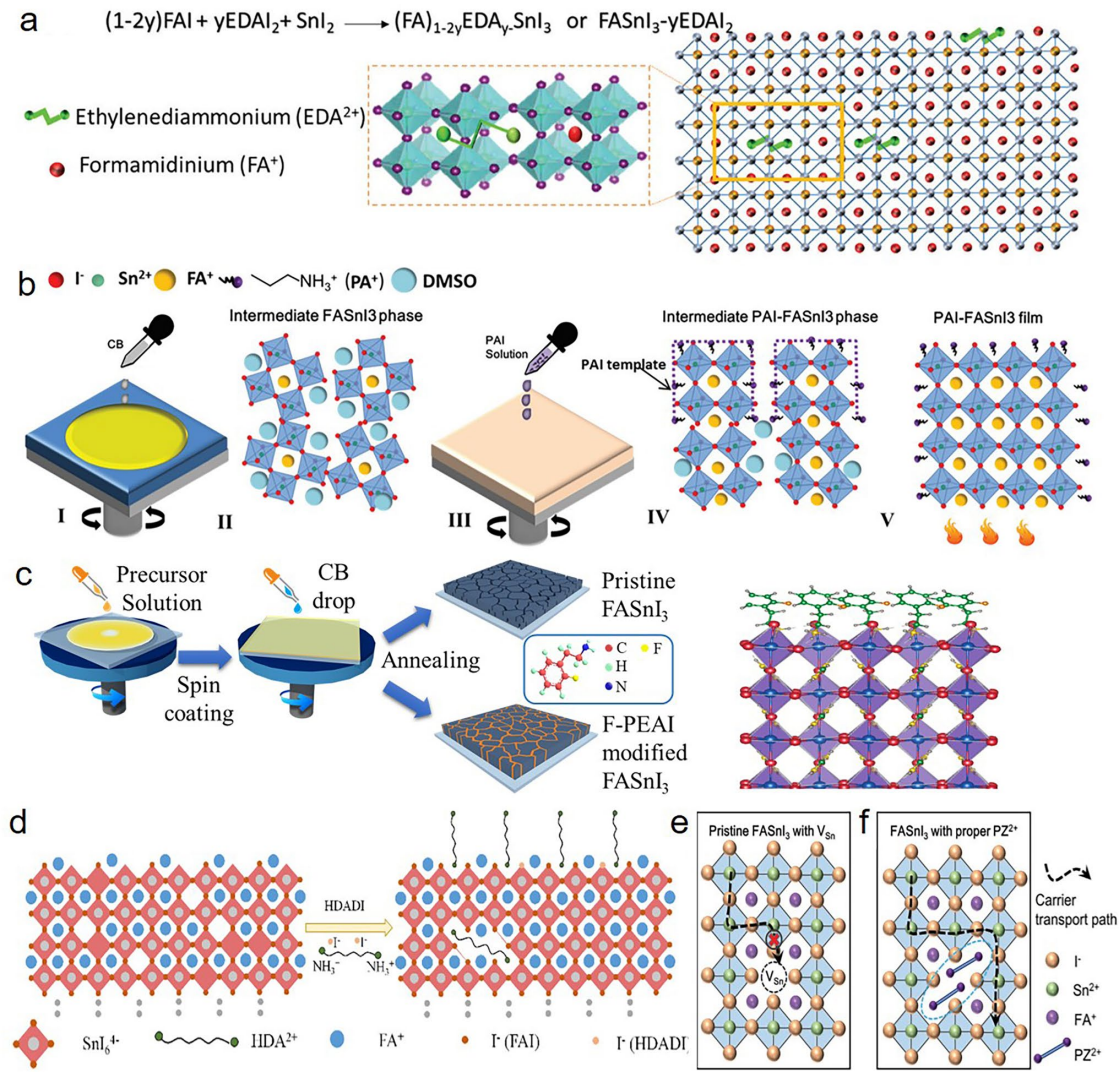
**a** Schematic representations of perovskite crystals in the presence of EDAI_2_ additives. Reproduced with permission from Ref. [59]. **b** Proposed scenarios of the templated growth of the TG-FASnI_3_ perovskite films. Reproduced with permission from Ref. [61]. **c** Schematic diagram of film preparation process, with the illustration of the 3D structure of 2-F-PEA, and DFT simulation of the steric arrangement of 2-F-PEAI. Reproduced with permission from Ref. [64]. **d** Schematic diagrams of FASnI_3_ perovskite crystallinity in the presence of the HDADI additive. Reproduced with permission from Ref. [68]. Schematic diagram of perovskite structures and carrier transport pathways for **e** pristine FASnI_3_ with V_Sn_, and **f** FASnI_3_ with proper PZ^2+^ content. Reproduced with permission from Ref. [71]

Large-volume amine ligands, such as phenylethylammonium (PEA) and butylammonium (BA), mainly applied to from the low-dimensionality to optimize their crystallization and enhance the stability. However, the introduction of some ligands with amine group directly optimizes the crystallization and passivate the bulk defects of Sn-based perovskites without forming low-dimensional structure. For example, Diau et al*.* incorporated ammonium salts ethylenediammonium diiodide (EDAI_2_) in the FASnI_3_ precursors to passivate defects and controlled the film morphology. They found that two ammonium functional groups enabled stronger interactions between EDA^2+^ cations and [SnI_6_]^4−^ units. Besides, the EDA^2+^ cation can occupy the two FA^+^ vacancies of the perovskite (Fig. 4a), leading to the reduction of defect states and the modification of the film morphology. Thus, they significantly decreased background carrier densities and increased the carrier lifetimes from 0.1 to 1.5 ns. Most importantly, the constrained EDA^2+^ cations could adjust their conformation to optimize crystal structure and leads to the lattice relaxation, which would cause a PCE increase from 6.3 to 8.9% after a storage for over 1400 h [59]. Similarly, Hayase et al*.* introduced ethane-1,2-diamine (edamine) by a simple post-treatment to passivate the dangling bonds and defects through bonding the under-coordinated tin with free electron pairs of the amine group. Such coordination resulted in the 50% enhancement of carrier life time and 0.1 V increase of the device *V*_*oc*_ [60]. In 2020, Han et al*.* employed the n-propylammonium iodide (PAI) in a mixed solvent of chloroform (CF) and DMSO (100:1 v/v) to induce the templated growth of FASnI_3_ crystals (TG-FASnI_3_) before annealing. As illustrated in Fig. 4b, DMSO could partially dissolve the crystals to provide a liquid phase environment for PAI to aggregate the newborn nucleus and form a templated growth of FASnI_3_ crystals along the (100) plane. Thus, the electron diffusion lengths were increased from 77 to 182 nm and defect density of the PAI–FASnI_3_ device (5.41 × 10^15^ cm^−3^) was one fifth of that in the FASI_3_ based device (2.89 × 10^16^ cm^−3^). As a result, a PCE of 11.78% with enhanced operation stability was obtained [61]. More recently, Zhao et al*.* employed and compared three ethylammonium halides, EAX (X = Cl, Br, I) to explore their roles in Sn-based perovskites. The result showed that crystallinity and orientation of perovskites are optimized by the regulation of EAI. Besides reduced defect density and enhanced crystallinity, the widest band gap was also obtained by employing Br^−^. Notably, Sn-based perovskites with EACl modification exhibited the best crystallinity, lowest defect density and excellent antioxidant capacity. They hold the opinion that most of Cl^−^ distribute on the surface and grain boundary to passivate defects, while a small amount of Cl^−^ enter the lattice to passivate I vacancy. The relevant PSC showed a PCE of 12.50% with enhanced operational and shelf stability [62].

Different from the single alkyl chain, benzene rings, with inherent hydrophobic and steric hindrance, have been demonstrated that could improve the stability of perovskite materials without inserting into the lattice. A more conjugated with polarizable backbone could facilitate photoexcited charge transport, which in turn lead to improve solar cell performance. Based on these benefits, Wu et al*.* designed a conjugated large-volume amine cation named 3-phenyl-2-propen-1-amine (PPA) as an additive to modify the FASnI_3_ film (note: PPA_x_FA_1−x_SnI_3_ is different from quasi-2D structure of PPA_2_FA_n−1_Sn_n_I_3n+1_). They found that PPAI would appear at the boundaries and thus enable the large-size grains with preferential orientation due to the Ostwald ripening. Moreover, the rigid conjugated structure (C_6_H_5_–CH=CH–) in PPAI deepened the VBM and CBM of FASnI_3_ film, which enabled the more favorable energy-level matching between the PEDOT: PPS layer and C_60_ layer, leading to the efficient charge extraction. The PPA-modified devices showed an interesting self-healing effect after heating or exposing to air, and the order of self-healing ability followed the same order of the molecule volume of PPA > PEA > BA, confirming that the self-healing effect could be attributed to the steric hindrance of the large-volume amines and be proportional to its volume. Consequently, the PPA-modified 3D FASnI_3_ device showed a PCE of 9.61% on 0.09 cm^2^ and 7.08% on 1 cm^2^ with robust stability and self-healing ability [63]. After that, Wu et al*.* also developed three fluorinated aniline organic ligands to achieve the simultaneous restriction of Sn^2+^ oxidation and regulation of crystallization. The corresponding scenario is illustrated in Fig. 4c. The result of density functional theory (DFT) calculation indicated that benefiting from the parallel distribution of the 2-F-PEA ligands at the surface of FASnI_3_ lattice, the chemical environment of the topmost Sn^2+^ was slightly different with pristine FASnI_3_, which indicated that the formation energy of Sn vacancy was increased. With the incorporation of the 2-F-PEAI, the concentration of deep defect states was reduced by 1–2 orders or magnitude [64]. In a recent study, Wu et al*.* prepared the high-quality film of FASnI_3_ perovskite via the introduction of trifluoroethylamine iodide (3FEAI) into the chlorobenzene (CB) solution. Different from the conventional ligand PEAI, the short-chain 3FEAI led to a smaller charge-exchanging resistance in perovskite bulk and interface of the absorber layer/transporting layer, reducing the non-radiative interface recombination, which allowed the improvement of *V*_*oc*_, *J*_*sc*_ and FF. Consequently, the champion device with 3FEAI modification showed a considerable PCE of 9.34% with long-term stability [65].

In addition, Han et al*.* developed that introducing pentafluorophen-oxyethylammonium iodide (FOEI) molecule with five fluorine atoms on benzene ring into the perovskite precursors could reduce the surface energy of the solution-air surface and optimize the crystal orientation. GIWAXS results showed a more preferred (h00) crystal orientation and the crystallization intensity of the FASnI_3_-FOEI perovskite films was significantly enhanced by 20-fold. Besides, FOEI could also passivate the iodide defects through the ionic bonding between the ammonium cation and iodide anion. Sn^2+^ was inhibited from reacting with water or oxygen and Sn^4+^ defects were reduced due to the hydrophobic nature of FOEI, which was confirmed by XPS measurements. Hence, a certificated efficiency of 10.16% based on FASnI_3_-FOEI perovskite films with high operational stability was obtained [66]. Similarly, Meng et al*.* introduced 2-phenoxyethylamine bromide (POEBr) to tune the surface energy of different facets of FASnI_3_ perovskite crystals, and thus obtained highly oriented FASnI_3_-POEBr perovskite films. The result of in situ ultraviolet–visible (UV–vis) absorption spectroscopy and in situ scanning electron microscopy (SEM) showed that the growth process of Sn-based perovskites in their system could not be explained by the classical Ostwald ripening (OR) mechanism. Then they proposed a crystal growth kinetics mechanism called “oriented attachment (OA)”, where two smaller nanocrystals with the same crystallographic orientation integrate to generate a larger nanocrystal, leading to the formation of oriented perovskites. Such unique mechanism offered Sn-based perovskites with lower density of defects and a higher PCE of 14.32% [67].

Huang et al*.* studied the effect of an organic cationic salt hexamethylenediamine diiodide (HDADI) on the crystallinity and morphology of FASnI_3_ perovskite. They found that the addition of 1% HDADI enabled the high-quality perovskite films with large coverage, high crystallinity, and disappeared pinholes as well as a prolonged carrier lifetime, which were associated with the NH_3_^+^ from HDADI interacting with iodide from [SnI_6_]^4−^ octahedra via a hydrogen bond (N–H···I) (Fig. 4d). This interaction not only neutralized charged defects or dangling bonds of perovskites but also formed a shield to retard the oxidation of Sn^2+^ to Sn^4+^ and reduce Sn vacancies. Also, the HDADI-doping FASnI_3_ acquired a champion PCE of 7.6% and an outstanding long-term stability of over 550 h to retain 80% of the initial efficiency in a glovebox with a N_2_ environment [68]. Hu et al*.* employed an aminoguanidine hydrochloride (NH_2_GACl) into Sn-based perovskite. The hydrogen bonding interaction between this ammonium end group and halide ions (N–H···I^−^) could passivate the defects and lessen the formation of Sn vacancies. Besides, the addition of NH_2_GACl significantly tuned the energy level of the perovskite layer to facilitate the charge transport. As a result, the PCE of the PEA_0.1_FA_0.9_SnI_3_ PSCs was improved from 4.72% to 7.3% after incorporating suitable amount of NH_2_GACl [69].

Chen et al*.* demonstrated that the π-conjugated polymer, poly[tetraphenylethene 3,3′-(((2,2-diphenylethene-1,1-diyl) bis(4,1-phenylene)) bis(oxy)) bis(N,N-dimethylpropan-1-amine) tetraphenylethene] (PTN-Br) passivated the defects of FASnI_3_ perovskite film and ensured excellent hole transportation. The formation of Lewis adducts between uncoordinated Sn atoms and the dimethylamino in PTN-Br reduced trap-assisted recombination and bimolecular recombination so as to enhance charge transportation [70]. More recently, large organic piperazine cations (PZ^2+^) were introduced by Yin et al*.* into the lattice of 3D FASnI_3_ perovskite to suppress the bulk defects, which was believed to be the largest organic cation that can enter 3D perovskite structure without reducing the dimensionality. As illustrated in Fig. 4e, due to the low formation energy, the ubiquitous bulk V_Sn_ defects act as the destroyer of the local [SnI_6_] inorganic structure and also the recombination center to capture carriers. Nevertheless, the modification of PZ^2+^ formed organic cation aggregation area with electrical neutrality instead of carrier capture center. FASnI_3_ perovskite with proper PZ^2+^ in Fig. 4f maintained the continuity of [SnI_6_] lattice and was beneficial for the carrier transport, which was different from low-dimensional perovskite with alternately arranged [SnI_6_] slabs. The FASnI_3_ PSC device with 1%PZ gained a PCE of 9.15%, mainly resulted from the reduction of the bulk defects [71].

Yang et al*.* disclosed pre-nucleation clusters (PNCs) to modulate the crystallization kinetics of FASnI_3_ through the introduction of the piperazine dihydriodide (PDI_2_). They found that PDI_2_ could tune the colloidal chemistry of the FASnI_3_ perovskite precursor solution to form a non-classical two-step nucleation, leading to the stable large clusters with low Gibbs free energy barrier, which accelerated the nucleation process and thus lowered trap density FASnI_3_ film. This pre-nucleation clusters assisted by the PDI_2_ enabled the control of the nucleation and crystal growth, resulting in a high-quality perovskite film with a longer TRPL lifetime (127.4 ns) than that of the control one (9.1 ns). Attributed to these benefits, a PCE of 11.39% with high long-term stability was obtained by the FASnI_3_-PNCs devices [72].

#### Coordination by Ligands with Hydroxyl Group

**Fig. 5:**
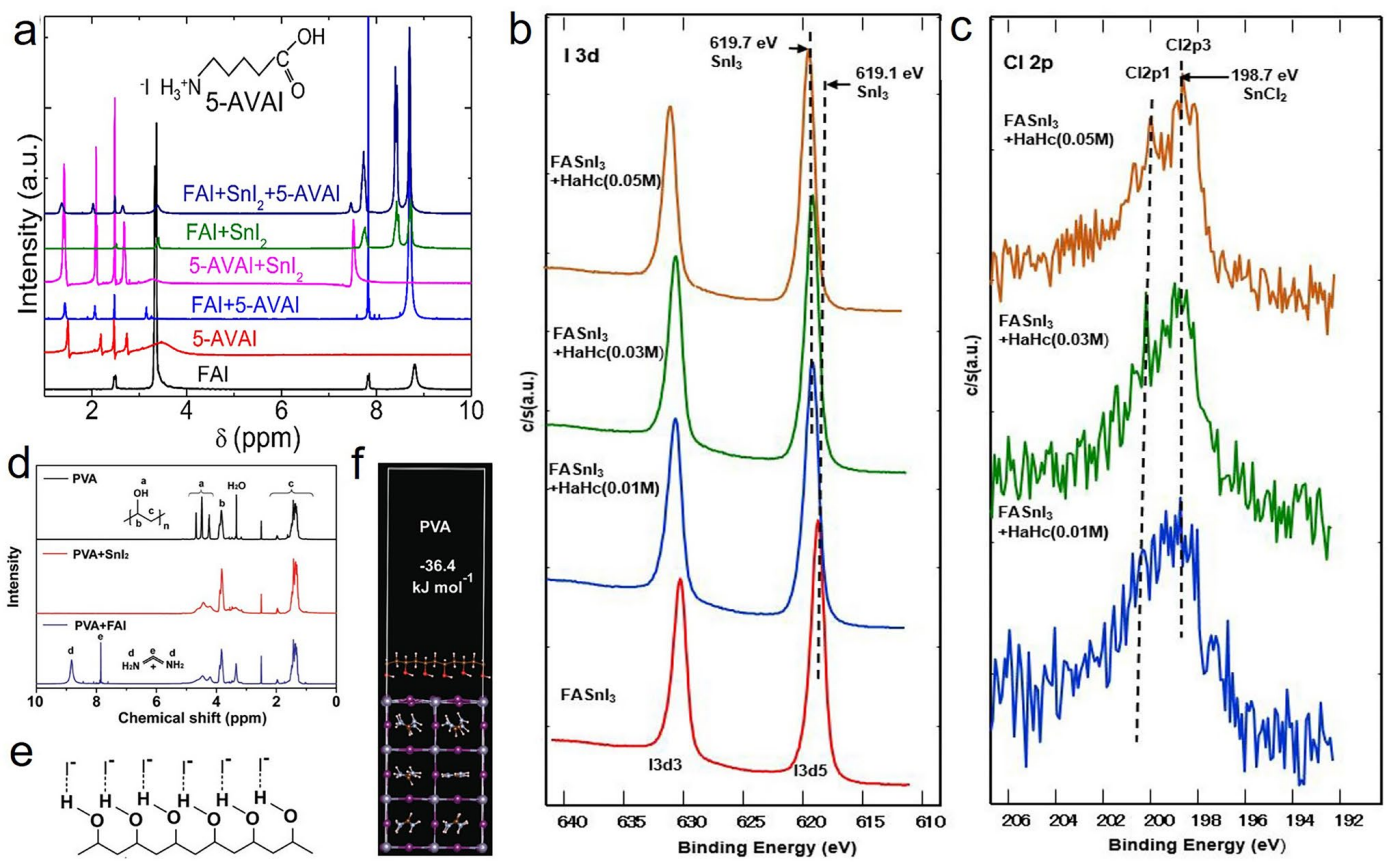
**a** ^1^H NMR spectra of FAI, 5-AVAI, FAI + 5-AVAI, 5-AVAI + SnI_2_, FAI + SnI_2_, and FAI + SnI_2_ + 5-AVAI in DMSO-*d*_*6*_ solution. Reproduced with permission from Ref. [73]. **b**–**c** High-resolution XPS spectra of I 3d and Cl 2p of FASnI_3_ without and with addition of various amount of HaHc. Reproduced with permission from Ref. [74]. **d** ^1^H NMR spectra of PVA, PVA + SnI_2_, and PVA + FAI in DMSO-*d*_*6*_ solution. **e** Representation of the O–H…I^−^ hydrogen bonding interaction. **f** DFT calculation of the absorption energy of PVA to the FASnI_3_ surface. Reproduced with permission from Ref. [75]

The additives with hydrogen bonding interaction play an effective role in improving the morphology and passivating grain boundaries of the tin perovskite films. Besides amino groups' function as hydrogen bond interaction, other types of hydrogen bonding were also investigated widely, especially the hydrogen bond achieved by the interaction between hydroxyl group and iodide ion in perovskite. For example, Islam et al*.*, studied the effects of carboxyl-functionalized 5-ammonium valeric acid (5-AVAI), which formed not only a hydrogen bond interaction (N–H···I^−^) by the ammonium end groups (–NH_3_^+^), but also an interaction by the carboxylic acid (–COOH) end groups (O–H···I^−^). The ^1^H NMR spectra in Fig. 5a confirmed that with the incorporation of 5-AVAI, a new proton resonance peak appeared at 7.5 ppm, which could be assigned to the coordination of functional groups of 5-AVAI with iodide ions. As a result, the pinhole-free homogeneous and stable film with one order of magnitude lower dark current density comparing with pristine sample was achieved. In addition, the introduction of 5-AVAI significantly improved the *V*_*oc*_ from 0.36 to 0.59 V, well the *J*_*sc*_ raised from 15.75 to 18.89 mA cm^−2^, and the PCE of PSCs improved from 3.4% to 7.0%. Moreover, highly stable PSCs exhibited a record of 100 h stability under 1 sun continuous illumination at maximum power point [73].

Later, the same group studied the implementation of hydroxylamine hydrochloride (HaHc) with FASnI_3_ perovskite. The results of first principal calculations showed that the structure of FASnI_3_ with hydroxylammonium (HA) ion forming O–H···I^−^ bond was stable than that without O–H···I^−^ bond, indicating such hydrogen bond would retard the volatilization of I^−^ anion, leading to the formation of stoichiometric FASnI_3_ perovskite. XPS spectra in Fig. 5b, c showed that the binding energies of I 3*d*5 shifted to higher energy positions compared to the pristine FASnI_3_ films, while the binding energies of Cl 2*p* from SnCl_2_ additive remained unchanged, which further confirmed the electronic passivation induced by the coordination with HaHc ligand. As the result, the relevant PSC with the inverted planar configuration gained the champion PCE of 9.18% with a light soaking stability for over 500 h [74].

Poly(vinyl alcohol) (PVA) with a dense hydroxyl group can create directional hydrogen bonding interactions that involve polar hydrogen groups O^δ−^–H^δ+^ and electronegative iodide ions I^δ−^. Han et al*.* employed PVA into the FASnI_3_ perovskite precursor solutions. As shown in Fig. 5d, once SnI_2_ or FAI was added into the PVA solutions, resonance signals of the –OH protons of PVA were obviously broadened, indicating the O–H···I^−^ hydrogen bonding interaction (Fig. 5e). DFT calculations also supported the results. The absorption energy of PVA to the FASnI_3_ surface, which was defined as $\Delta \mathrm{E}=\mathrm{E}\left[{\mathrm{FASnI}}_{3}-\mathrm{PVA}\right]-\mathrm{E}\left[{\mathrm{FASnI}}_{3}\right]-\mathrm{E}\left[\mathrm{PVA}\right]$, was calculated to be − 36.4 kJ mol^−1^. The optimized structure after the energy relaxation is shown in Fig. 5f. The O–H···I^−^ hydrogen bonding interactions between PVA and [SnI_6_]^4−^ lattice have the ability to introduce nucleation sites, slow crystal growth, guide crystal orientation, reduce trap states and inhibit iodide migration. The FASnI_3_–PVA PSCs attained higher PCE of 8.9% with significantly improved *V*_*oc*_ from 0.55 to 0.63 V. More importantly, the FASnI_3_–PVA PSCs exhibited remarkable long-term stability, with no decay in efficiency after 400 h of operation at the maximum power point [75]

### Ligands for Coordination with Sn Cations

In ligand-assisted pathway, ligand with functional groups with lone pair electrons such as carbonyl group (C=O) shows a strong passivation effect on the under-coordinated Sn^2+^ cation via forming an intermediate phase in perovskite framework, which also retards the crystallization rate to obtain dense and uniform film with lower defect density. Such Lewis acid–base coordination was widely proved to be effective due to the strong Lewis acidity of Sn^2+^.

**Fig. 6:**
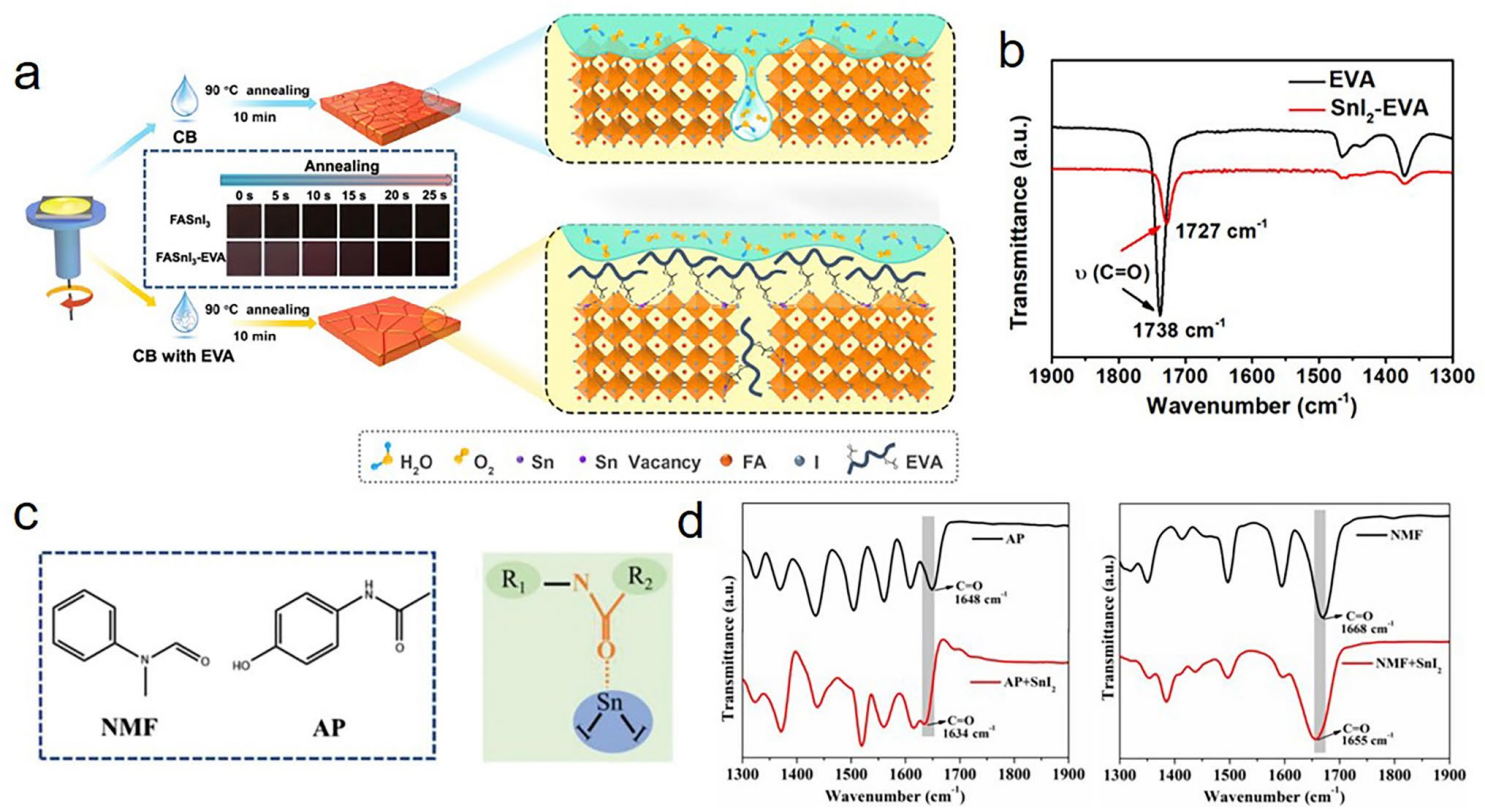
**a** Schematic illustration of the preparation process of Sn-based perovskite films with and without EVA treatment, respectively. **b** FTIR spectra of pure EVA and SnI_2_–EVA. Reproduced with permission from Ref. [76]. **c** Molecular structures of NMF and AP, schematic illustration of the interaction between the additive molecule and SnI_2_. **d** FTIR of pure AP and the complex of AP–SnI_2_, and pure NMF and the complex of NMF–SnI_2_. Reproduced with permission from Ref. [78]

Chen et al*.* introduced a unique polymer [poly (ethylene-co-vinyl acetate)] (EVA) into antisolvent during spin-coating of FASnI_3_ precursor solution (Fig. 6a). According to FTIR spectra in Fig. 6b, the stretching vibration of the carbonyl bond in EVA shifted to a lower wavenumber in the EVA–SnI_2_ composite, confirming the chemical interaction of EVA with SnI_2_. The powerful Lewis acid–base complexation between C=O groups in EVA and uncoordinated Sn not only greatly retarded the crystallization rate and reduced the generation of films defects, but also possessed a self-encapsulation effect that could effectively prevent perovskite from being destroyed by moisture and oxygen. Consequently, the perovskite films with a stronger PL intensity and longer lifetime were obtained. The EVA-modified FASnI_3_ device exhibited a PCE of 7.72% with excellent environmental stability in high-humidity air [76].

Yin et al*.* introduced the thiosemicarbazide (TSC) to modulate defect state density at surfaces and grain boundaries in CsSnI_3_ perovskites. The functional group S=C–N with strong electrostatic attraction in TSC could make strong coordination interaction with Sn ion, and the TSC were inserted into the SnI_2_ interlayer and anchored on SnI_2_. After the annealing, the TSC molecules anchored on the surface and grain boundary, leading to the further passivation of the Sn-related defects. After incorporating the TSC passivator, the proportion of the decay lifetime obviously decreases from 62.3% (pristine) to 27.0% (TSC), and the lifetime constant increased from 9.6 (pristine) to 39.1 (TSC) ns, respectively. Especially, an obvious *V*_*oc*_ enhancement from 0.47 to 0.63 V is due to the reduced deep level trap-state density with the surface passivation of TSC [77]. Wu et al*.* compared the introduction of *N*-Methylformanilide (NMF) and 4-acetamidophenol (AP), which both owned the functional group of O=C–N (Fig. 6c). As depicted in Fig. 6d, the shift of stretching vibration peaks of carbonyl bond (C=O) in FTIR spectroscopy for both NMF–SnI_2_ and AP–SnI_2_ samples indicated the interaction with SnI_2_, which slowed down the SnI_2_ dissociation, and resulted in retard of the crystallization and suppression of Sn vacancy. Especially, the reductive property of AP further strengthened the effect against Sn^2+^ oxidation. As a result, the champion device obtained a PCE of 10.03% and long-term stability for over 1000 h [78]. In addition, they introduced a multifunctional medium trifluoroacetamide (TFA). The amide group (–CONH_2_) could achieve the coordination of C=O···Sn^2+^ and NH_2_···I^−^ simultaneously, and thus perovskite film with highly ordered crystallization orientation and low defect density was obtained [79]. Recently, Wu et al*.* introduced a conjugated non-fullerene molecule (IO–4Cl) with n-type semiconductor property into Sn-based perovskites, where the C=O group could establish strong bonds with Sn^2+^ to regulate grain growth and passivate defects. Besides, due to the appropriate lowest unoccupied molecular orbital level and interface modification ability, IO–4Cl enabled superior electron extraction and transport ability of Sn-based perovskites [80].

Besides, Han et al*.* introduced the 4-fluorobenzohydrazide (FBH) into antisolvent CB to form a carbonylate antioxidant capping layer atop the perovskite film. FTIR spectroscopy demonstrated that the signal of C=O bond for FBH shifted to a lower wavenumber for the mixture of FBH–SnI_2_, which was in similar with aforementioned studies, indicating the coordination between C=O group in FBH and Sn^2+^. This coordination effect changed the morphology of the FASnI_3_ film and reduced the defects. Compared with the control sample, the FBH-treated sample exhibited a strong PL intensity and the carrier lifetime just depicted a slight drop from 4.58 to 4.15 ns when the oxygen content rose up from 0.1 to 100 ppm. As a result, a champion efficiency of 9.47% under normal conditions (0.1 ppm oxygen) and 9.03% at a high oxygen level (100 ppm oxygen) with excellent light stability were obtained [81].

Han et al*.* synthesized three π-conjugated Lewis base molecules with different structures, namely 2-cyano-3-[5-(2,4-difluorophenyl)-2-thienyl]-propenoic acid (CTA-F), 2-cyano-3-[5-(2,4-dimethoxyphenyl)-2-thienyl]-propenoic acid (CTA-OMe), and 2-cyano-3-[5-[4-(diphenylamino)phenyl]-2-thienyl]-propenoic acid (CDTA). All the molecules could form intermediate phase through the interaction of C=O bond and C≡N bond with the Sn^2+^ cations, leading to compact and uniform perovskite films with large increase of the carrier lifetime. More importantly, the electron-donating effect of triphenylamine unit in CDTA caused a stronger electron delocalization from the π-conjugated system to the Lewis base groups, which significantly increased the binding strength between CDTA and the Sn^2+^ cations. These benefits contributed to a stabilized PCE of 10.1% for the TPSCs treated with CDTA, and a certified steady-state efficiency of 9.2% was also obtained. Furthermore, the CDTA-treated device remained over 90% of its initial PCE after light soaking for 1000 h in air [82]. In addition, Chen et al*.* proposed a self-assembly molecule fluorinated-perylene diimide (F-PDI) to provide a structural framework for crystal growth and charge transfer. The interaction of C=O with Sn^2+^ and F with FA^+^ between F-PDI and perovskite could simultaneously passivate the surface defects and slow down the growth of perovskite crystals. Besides, the perovskite components could be effectively driven to the vertically orientated growth of perovskite crystals due to the floating self-assembly of F-PDI, which greatly promotes the effective transmission of intergrain carriers. Consequently, these favorable factors conduced to a high PCE of 9.49% with robust device stability. More importantly, the self-assembly behavior endowed the interface with excellent intrinsic hydrophobic property, which effectively prevented the perovskite film from the attack of moisture and oxygen [83]. Recently, a bi-linkable reductive cation, formamide (FM), was proposed by Chen et al*.* The –NH_2_ and C=O groups in the ligand could coordinate with FA^+^ and Sn^2+^ simultaneously, resulting in the enlarged colloidal size and optimized crystallinity [84].

Yin et al*.* introduced polyethylene glycol (PEG) polymer with plenty of ether bond groups (C–O–C) in a FASnI_3_ precursor to fabricate uniform and fully covered perovskite films with lower defect density. They proved that the hydrogen bond interactions between FA^+^ and C–O–C and the complexation through uncoordinated Sn with C–O–C could effectively regulate film crystallization and reduce defect state density. In this way, the PEG-modified FASnI_3_ devices exhibited a PCE of 7.53% and maintained 90% of initial PCE after 720 h of storage in a N_2_ glovebox [85]. The effect of ether group was also studied by Cho and co-workers. They employed a multifunctional molecular fulleropyrrolidine with a triethylene glycol monoethyl ether side chain (PTEG-1). The ether group (C–O–C) and fullerene group would interact with Sn^2+^ and I^−^, respectively, which suppressed the formation of Sn^4+^ and I_3_^−^. Meanwhile, the PTEG-1 ligand was found coexisting on both grain boundaries and surfaces and thus serves as an electron transport material to promote electron extraction [86].

Chen et al*.* employed graphite phase-C_3_N_4_ (g-C_3_N_4_) into the flexible tin-based PSCs. They found that the interaction of the hydrogen bond between the nitrogen (N) atoms in g-C_3_N_4_ and FA^+^ cation could slow down the crystallization rate. Meanwhile, the distance matching between the two binding sites (7.13 Å) and the lattice size of FASnI_3_ (6.33 Å) could enhance the passivation effect. Attributed to crystallographic size-effect, the promotional effect of g-C_3_N_4_ on flexible devices was superior than that on rigid devices, and a flexible tin-based PSCs with g-C_3_N_4_ realized a stabilized PCE of 8.56% with negligible hysteresis was achieved [87]. Seok et al*.* introduced 2-thiophenemethylammonium iodine (ThMAI) to investigate the dual effects on residual strain and surface passivation in Sn perovskite films. They found that thiophene units in ThMAI can interact with corner-sharing [SnI_6_]^4−^ octahedra through the Sn–S interactions, thus forming strong diploes with Sn atoms. Moreover, the Fermi level shifted by approximately 80 meV toward the CBM in the ThMAI-treated sample. Besides, the relaxation of the compressive strain in the Sn-based perovskite film leads the interplanar spacing after the post-treatment by ThMAI. Owing to these benefits, a record PCE of 9.06% of Cs_0.1_FA_0.9_SnI_3_ perovskite device was achieved [88].

Chen et al*.* introduced the 8-hydroxyquinoline (8-HQ) bidentate ligand to suppress the oxidation of Sn^2+^ to Sn^4+^ and improve the quality of FASnI_3_ films. Because the N and O atoms in 8-HQ could simultaneously coordinate with Sn^2+^ to form a relatively stable complex, the amount of Sn^4+^ decreased from 43.16% to 13.92% comparing with the control FASnI_3_ film. In addition, the improvements in PL peak intensity and carrier lifetime implied that the 8-HQ could suppress the non-radiative recombination. As a consequence, the 8-HQ treated device achieved an excellent PCE of 7.15% with improved N_2_ and air stability [89]. Hao et al*.* employed melamine to the perovskite precursor solution to modulate the crystallization and defects. By Lewis acid–base adduction and hydrogen bonding, the C=N and –NH_2_ functional groups in melamine simultaneously acted on Sn^2+^. Due to the high symmetry of molecular structure of melamine, the uniform potential distribution could facilitate the adduction with SnI_2_ in the precursor solution. Such interactions inhibited the oxidation of Sn^2+^ effectively. Meanwhile, clusters with larger colloid size would be helpful to promote the formation of larger perovskite grains. As the result, the collection and transport of carriers of the Sn-based perovskite film was promoted because of the larger grain size, and an enhancement of 100 mV in *V*_*oc*_ in target PSC was obtained [90].

Thiourea utilized as versatile ligands for Sn-based perovskite was studied by Mi et al*.* recently. By comparing the structural stabilities of FASnI_3_ with FAPbI_3_, it could find that FASnI_3_ adopted a stable perovskite structure while FAPbI_3_ spontaneously adopted a phase transition toward a yellow hexagonal phase under 400 K, which is against the theory that FASnI_3_ (tolerance factor *t* = 1.00) should be more easily to transform from the perovskite structure than FAPbI_3_ (*t* = 0.99). They tried to explain the conflict between experiment and theory by proposing that the interaction between Sn^2+^ and I^−^ in FASnI_3_ is stronger and more directional than that between Pb^2+^ and I^−^ in FAPbI_3_. Therefore, the strong interaction in FASnI_3_ precursor will result in the coordination of SnI_3_^−^ units with DMF or DMSO solvents, inducing the rapid crystallization of FASnI_3_. Meanwhile, the annealing process will remove the solvents and further cause more surface vacancies. Sulfur ligands with stronger Lewis basicities than their carbonyl counterparts were investigated. It was found that thiourea ligand *N*,*N*′-dimethylethylenethiourea (DMETU) can effectively compete with I^−^ for coordination with Sn^2+^ and simultaneously ligate with two adjacent Sn^2+^ centers. Such ligand would not be completely removed by annealing and could slow down the crystallization process, and thus protect the film surfaces. As a result, the DMETU ligand-modified PSC with the inverted device structure gained a maximum PCE of 12.3%, and retained 85% of its initial efficiency when being exposed to humid air without encapsulation [91].

Recently, ionic liquids had made a remarkable effect on lead-based perovskite, especially in regulating crystallization due to the carboxyl containing C=O group in ionic liquids forms strong coordination with tin atoms. Ionic liquid methylammonium acetate (MAAc), for instance, was introduced by Huang et al*.* as a mixture with DMSO to form low-dimensional Ruddlesden-Popper (LDRP) Sn-based perovskite BA_2_MA_3_Sn_4_I_13_. The addition of MAAc could help to form the intermediate BAMASn-Ac, which would produce dense BAMASn-I perovskite films by ion exchange between I^−^ and Ac^−^ [92]. Wu et al*.* prepared FASnI_3_-based PSCs with the addition of solid-state ionic liquids formamidine acetate (FAAc). The cation of FAAc can passivate the vacancy of FA^+^ in the crystallization process without introducing impurity cations. The coordination between the anion CH_3_COO^−^ (C=O group) with under-coordinated Sn atoms led to the formation of intermediate phase, which could slower the nucleation rate of FASnI_3_ grains, thus contributing to the high crystallinity perovskite film with large grain sizes and low trap density of states. As a result, 5 mol% FAAc-modified devices exhibited a champion PCE of 9.96% with long-term stability [93]. Hao et al*.* also studied the effect of FAAc comparing with acetic acid (HAc) and MAAc. The result showed that the coordination of C=O and Sn^2+^ from FAAc was stronger than that from HAc and MAAc. Moreover, FAAc could be beneficial in forming clusters with larger colloid sizes in precursor solution and thus reduce the nucleation density and slow down the crystallization rate [94].

Similarly, Abate et al*.* introduced the ionic liquid n-butylammonium acetate (BAAc) to adjust the precursor coordination and to control perovskite crystallization toward high-quality films. The solid O···Sn bonds were formed via chelation between Ac^−^ (CH_3_COO^−^) and Sn^2+^, while the N–H···X hydrogen bonds were established through interactions between the BA^+^ and I^−^/Br^−^ anions, which led to a stable precursor solution with retarded Sn^2+^ oxidation. As the formation of the perovskite crystals, BAAc would move to the grain boundaries and work as a bridge to eliminate the pinholes. Besides, the long chain BA^+^ cations are eventually expelled to the perovskite surface, resulting in excellent hydrophobicity and antioxidant properties of the perovskite. As a consequence, the preferentially oriented perovskite film with a lower amount of Sn (IV) and a high PCE of 10.4% were achieved, and BAAc-modified perovskite films possessed a stable crystal structure at 85 °C [95]. Chen et al*.* first applied the Ostwald ripening effect induced by 1-butyl-3-methylimidazolium bromide (BMIBr) ionic liquids to the fabrication of tin-based PVSCs. During the thermo-annealing of perovskite films, the tin-based perovskite precursor composites and part of the precipitated black perovskite could be dissolved by BMIBr due to its naturally strong polar and low melting point properties. As a result, the larger perovskite grains with lower chemical potential grow further with time, while the smaller perovskite grains disappeared, and the corresponding average grain sizes increased from 504 to 829 nm. Meanwhile, the carrier lifetime increases to 7.78 ns compared with the pristine perovskite film (4.96 ns). Consequently, the average PCE of the BMIBr-treated FASnI_3_ device increased from 7.22% to 9.63% [96].

## Effect of Ligands on Dimensional Engineering

Among all the structures created through ligand engineering, low-dimensional Sn-based perovskites have become one of the most promising research scopes. Low-dimensional perovskites are defined as structures that can conceptually be derived from specific slices of the 3D structure. The common perovskite layer consists of [Sn_n_I_3n+1_]^(n+1)−^ layers of corner-sharing octahedra, connected by monovalent or divalent organic cations. Aforementioned organic cations generally contain one or more terminal cation groups, which can interact with inorganic anions and effectively form hydrogen bonds, rather than halide compounds that interfere with inorganic thin films in space [98]. A large number of reports on Pb-based perovskite have proved the effectiveness of the low-dimensional structure [99, 100]. The introduction of large-size organic cations would suppress ion migration and molecule penetration, and also reduce self-doping concentrations. Meanwhile, the hydrophobicity of organic spacers can result in obvious enhancement of structural stability and moisture resistance [101, 102]. It should be noticed that despite the improved stability, the band gaps of 2D perovskites with single unit cell layer (*n* = 1, A_2_SnI_4_, A = bulky alkylammonium cations) are between 1.90 and 2.40 eV, which are much larger than those of their 3D analogues, and beyond the optical range of 0.9–1.6 eV for solar cells [103]. Moreover, due to the quantum confinement effect introduced by reduced dimensionality, the separation of photoexcited electron–hole pairs becomes difficult [104, 105]. Hence, the natural strategies would be to increase unit cell layer thickness (n) to form 2D and quasi-2D structure, or alternatively, to combine 3D layer and low-dimensional layer, forming heterojunction structure.

### Ligands for Forming 2D and Quasi-2D Structure

**Fig. 7:**
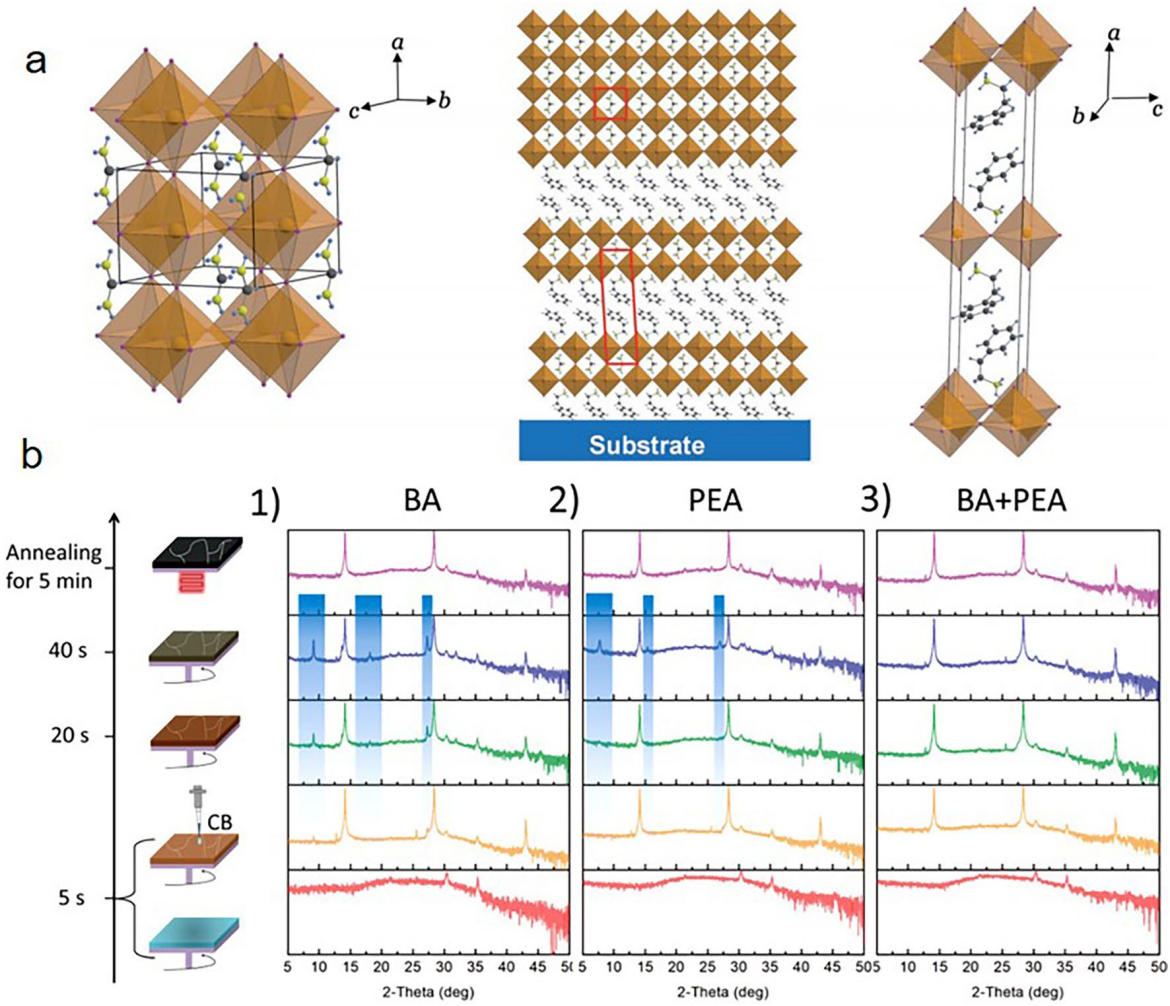
**a** Schematic crystal structure of 3D reference FASnI_3_, 2D/3D mixture (2D 0.08 M), with the unit cells of each component outlined in red, and 2D PEA_2_SnI_4_. Reproduced with permission from Ref. [109]. **b** Evolution of XRD patterns of 2D RP Sn-based perovskites depending on the film fabrication process. Reproduced with permission from Ref. [118]

The halide perovskites are dominated by the Ruddlesden-Popper (RP) archetypes, which are characterized by two offsets layers per unit cell, having pairs of interdigitated interlayer spacers [106]. In 2017, Kanatzidis et al*.* managed to fabricate 2D RP (CH_3_(CH_2_)_3_NH_3_)_2_(CH_3_NH_3_)_n−1_Sn_n_I_3n+1_ perovskite solar cell using a simple one-step spin-coating method. The optical band gaps decreased from 1.83 eV for *n* = 1 to 1.20 eV for *n* = $\infty$, among which the *n* = 3 and *n* = 4 perovskites owned band gaps of 1.50 and 1.42 eV. It was interestingly found that the slabs of [(CH_3_NH_3_)_n−1_Sn_n_I_3n+1_]^2−^ would parallel to the substrate when DMSO is used as solvent; Meanwhile, the slabs would become perpendicular as DMF works as solvent. The perpendicular arrangement for perovskite slabs was beneficial for carrier transport along I–Sn–I bonds, and the corresponding PSC showed a PCE of 2.5% when *n* = 4 [107]. Later, Ning et al*.* fabricated low-dimensional Sn-based perovskites by incorporating PEA spacers. With the increase amount of PEA, (020) facet of (PEA)_2_SnI_4_ could be observed in X-ray diffraction (XRD) spectra. Grazing-incidence wide-angle X-ray scattering (GIWAXS) was performed to prove the highly oriented perovskite film perpendicular to the substrate. The corresponding PSC exhibited the highest PCE of 5.94% with enhanced stability over 100 h [108]. After that, Loi et al*.* also investigated low-dimensional Sn-based perovskite by incorporating PEA ligand. By mixing a very small amount (0.08 M) of layered (2D) Sn perovskite with 0.92 M (3D) FASnI_3_, superior crystallinity and well-defined orientation of FASnI_3_ grains were induced. It could be observed in the XRD pattern that the first peak of the 2D/3D perovskite at 2$\theta$=3.8$^\circ$ indicated an *a*-axis of ~ 23 Å (Fig. 7a). According to the reported results, a double layer of PEA molecules occupies approximately 10.0 Å in the *a*-direction; meanwhile, a single layer of SnI_6_ octahedra occupies 6.3–6.4 Å. Therefore, it could be concluded that part of the 2D/3D film comprised of double layers of SnI_6_ octahedra connected with double layers of PEA molecules. As a result, a PCE of 9.0% in a planar p-i-n device structure was achieved. The PSC also showed considerable improved stability due to the 2D/3D structure [109]. Similarly, Lee and co-workers showed that the binary additives of PEAI and EDAI_2_ could play a role in reducing the dimensionality of the FASnI_3_ crystals from 3D to mixed 2D/3D. Hence, the film crystallinity and plane orientation are improved, resulting in better PSCs performance [110]. Later, Abate et al*.* also suggested that with the assistance of PEACl, more ordered and vertically oriented 2D Sn-based perovskite crystals were enabled. In addition, PEACl would act as a barrier layer at the surface of the crystals, thus protecting the active layer from oxidation [111]. The halogen engineering that partially substitutes PEAI with PEABr could improve the structural stability and the charge transfer ability. Hao et al*.* obtained 2D/3D Sn-based perovskite with reduced residual strain along the (*h*00) planes and improved crystallinity by the introduction of PEABr [112].

The function of formamidinium thiocyanate (FASCN) in quasi-2D Sn-based perovskite is proved by Kim and co-workers. FASCN is beneficial for the coarser perovskite grain and higher degree of crystallinity in the out-of-plane direction. The PEAI ligand incorporated perovskite solar cell showed a PCE of 8.17% along with a steady-state PCE of 7.84% at maximum power point (MPP) [113]. Nazeeruddin et al*.* found the use of symmetrical imidazolium-based cations, such as benzimidazolium (Bn) and benzodiimidazolium (Bdi), would allow the formation of 2D perovskites with relatively narrow band gaps compared to traditional –NH_3_^+^ amino groups. 2D perovskite Bn_2_SnI_4_ showed an optical band gap value of 1.81 eV, while BdiSnI_4_ showed the value of 1.79 eV. PSC based on Bn_2_SnI_4_ was fabricated and the corresponding PCE reached 2.3%, with a steady-state power output at maximum power point over several minutes. This work demonstrated that 2D imidazolium-based tin perovskite is promising because of the suitable bandgap and superior stability [114]. In the field of MASnI_3_, thiophene-based 2-thiophene-ethylammonium iodide (TEAI) was utilized as the spacer cation and quasi-2D layered perovskite was obtained. XRD pattern exhibited that when the proportion of TEAI is increased to 40%, diffraction peaks below 10$^\circ$ could be observed, indicating the presence of low-number 2D structure (n < 4). As a result, the PSCs showed a PCE of 6.8%, which was a considerable result for MASnI_3_-based solar cells [115].

Recently, He et al*.* achieved a remarkable PCE of 14.81% by employing indene-C_60_ bisadduct (ICBA) as electron transport layer (ETL) and 4-fluoro-phenethylammonium bromide (FPEABr) in the perovskite precursor solution. 2D phase was believed to induce highly oriented 3D FASnI_3_ and was revealed that mainly located at the top and bottom surfaces of the film, as well as 3D grain boundaries. Benefiting from this unique microstructure, the oxidation of Sn is significantly suppressed, while the defect density is reduced, thereby improving the device performance [116].

**Fig. 8:**
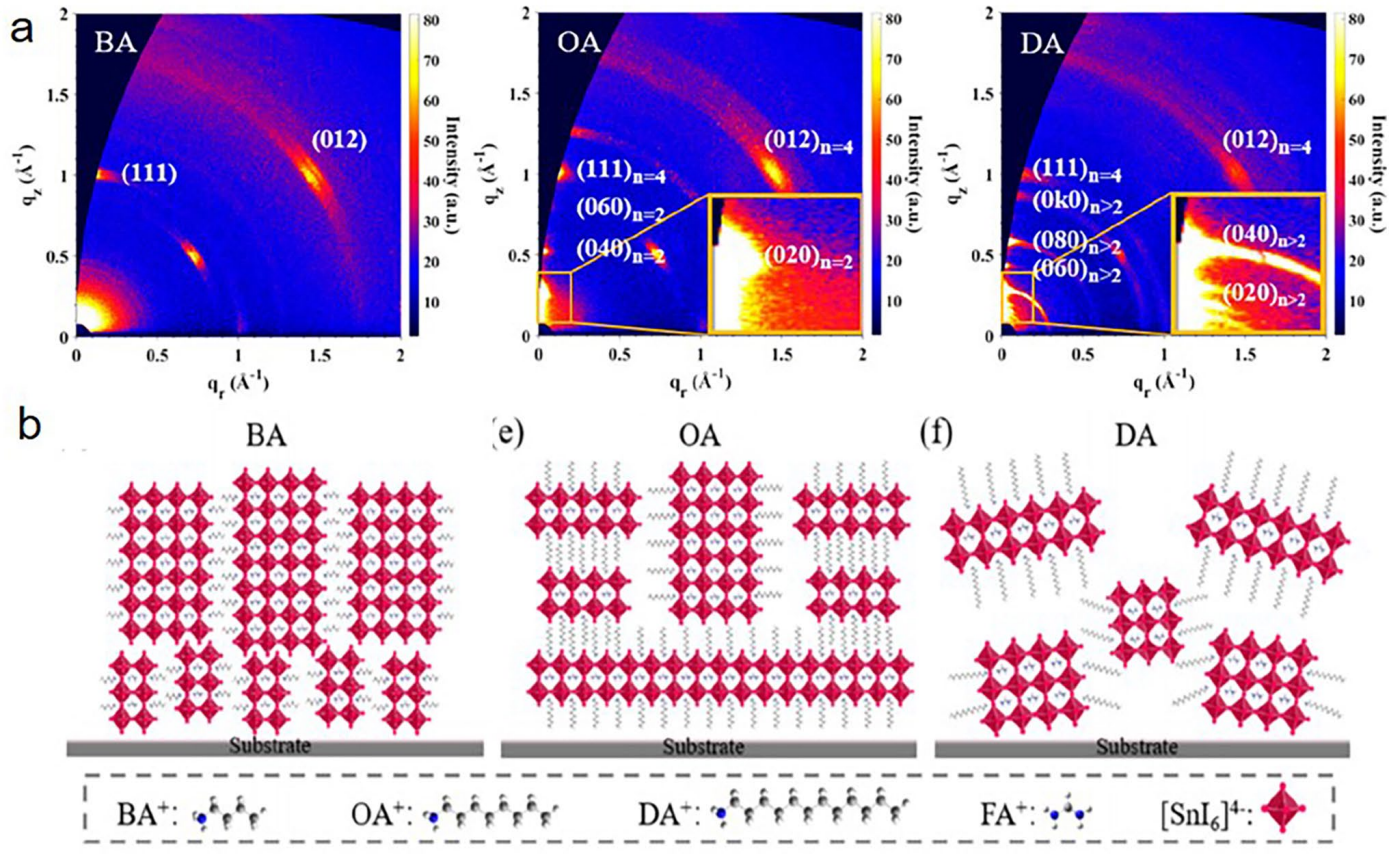
**a** GIWAXS images of 2D perovskite films based on BA, OA, and DA. **b** Schematic illustration of crystal orientation, dimensionality, and phase distribution of BA, OA, DA-based 2D perovskite films. Reproduced with permission from Ref. [121]

Expect for aromatic ligands, butylammonium (BA), for instance, was studied in hybrid perovskite BA_2_MA_n−1_Sn_n_I_3n+1_ (*n* = 2–4). By increasing the layer thickness from *n* = 1 to 4, the band gap decreased from 2.04 to 1.75 eV. The smaller carrier effective mass, strong exciton effects and better light absorption for BA-introduced 2D hybrid perovskite are highly desirable for the design of PSCs with reasonable performance and greatly enhanced device longevity [104, 117]. Huang et al*.*, in 2019, first introduced BA^+^ and PEA^+^ ligands simultaneously to control the crystallization of 2D RP Sn-based perovskite films. XRD pattern in Fig. 7b showed 2D RP Sn-based perovskite films at different time points during crystal growth. It could be found that diffraction peaks at 2$\theta$ = 14.12$^\circ$ and 28.32$^\circ$ in three systems appeared immediately after the deposition of antisolvent, corresponding to (111) and (202) planes of Sn perovskites. Moreover, some additional peaks appeared in BA system and PEA system (blue gradient columns in Fig. 7b). These additional peaks were confirmed representing 2D perovskite (*n* = 1) intermediate phases, which could impede the growth of main of the RP phases significantly, resulting in uneven nucleation and disordered orientation. On the contrary, these peaks were not found in BA + PEA system, demonstrating that the intermediate phases were not formed through the co-work of mixed spacer cations. Such effect could be helpful in forming smooth, highly oriented films with fewer bulk defects and surface traps [118]. Besides BA, 5-ammonium valeric acid (5-AVA^+^) ligand was introduced as organic spacer by Chen et al*.* with NH_4_Cl as additive. Highly vertically oriented quasi-2D Sn-based perovskite AVA_2_FA_n−1_Sn_n_I_3n+1_ (*n* = 5) was employed as light absorber and gained a PCE of 8.71% [119]. Loi et al*.* incorporated ethylammonium iodide (EAI) into 2D/3D Sn-based perovskite (where 2D is PEA_2_FASn_2_I_7_), and thus optimized FASnI_3_ grains with increased size and preferred orientation in the out-of-plane direction was obtained. These changes further lead to much lower trap density, background charge carrier density and charge recombination loss in EA_x_2D/3D-based PSCs [120]. In 2020, Liu et al*.* compared the effects of alkyl chain length on crystal growth and oxidation process in two-dimensional Sn-based perovskites. They applied alkylamines spacer cations with different alkyl chain lengths: butylamine (BA), octylamine (OA), and dodecylamine (DA). By combining GIWAXS with PL spectra, they came the conclusion that the organic spacer cations with shorter chain length are more favorable to induce oriented crystal growth and ordered phase distribution (Fig. 8a). Longer alkyl chains promote parallel crystal growth of 2D Sn-based perovskite films, while shorter chain facilitates perpendicular crystal growth (Fig. 8b) [121]. Inspired by the function of the alkylammonium (ALA, CH_2_=CH_2_CH_3_NH_3_^+^) in suppressing the formation of narrow quantum wells and extending the carrier diffusion lengths in Pb-based perovskites, Liu et al*.* gained quasi-2D Sn-based perovskite through ALA cations. GIWAXS patterns indicated that ALA cations were able to induce an in-plane alignment of the (*h*00) crystal planes at room temperature, which reduced the randomness in crystal orientation and facilitated charge carrier transport [122].

**Fig. 9:**
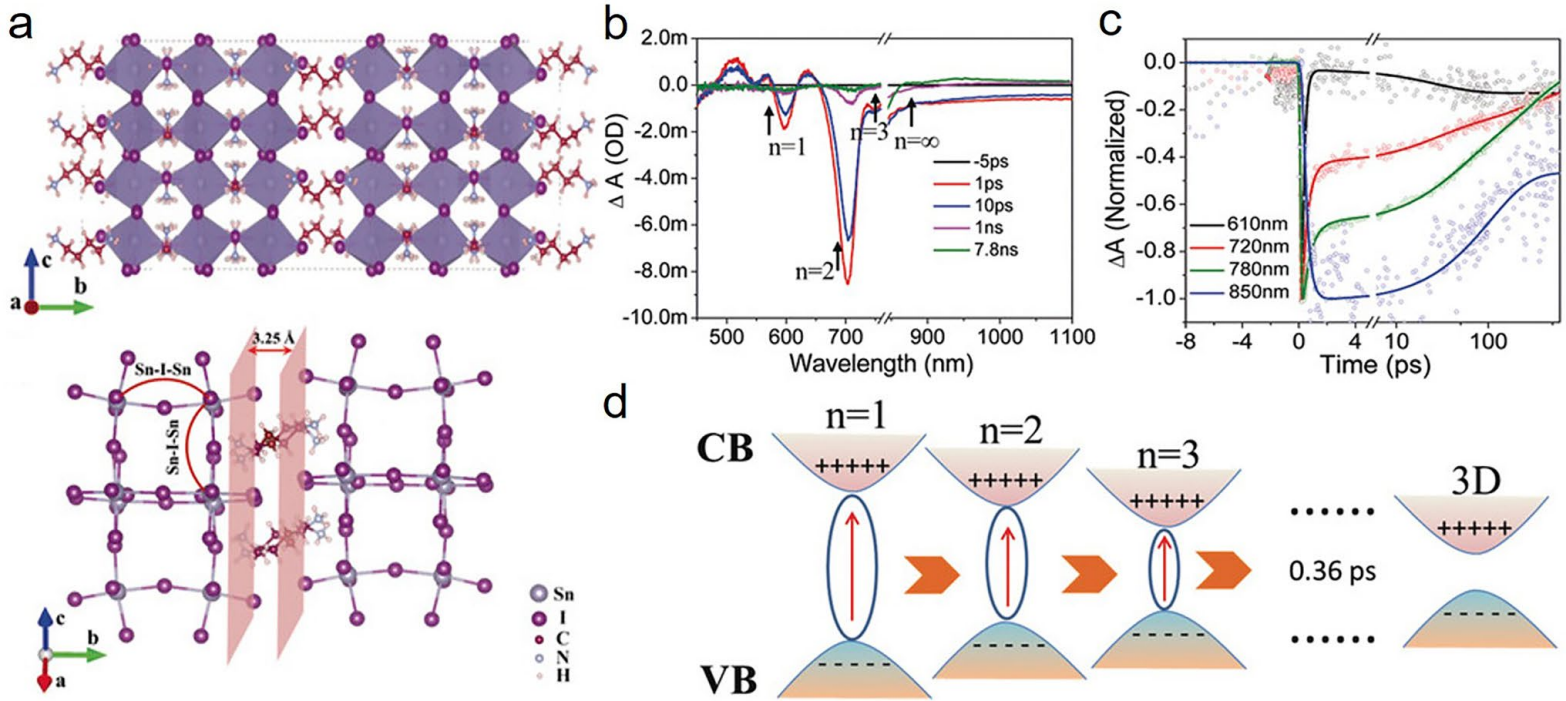
**a** Crystal structures of the 2D perovskite (BEA)(FA)_2_Sn_3_I_10_ and Illustration of distance of respective diffraction planes. **b** TA spectra at various delay times for (BEA)FA_2_Sn_3_I_10_ film. **c** TA kinetics probed at *n* = 1, 2, 3 and n $\approx \infty$ bands. **d** The band structure for mixed perovskite QWs and carrier transport pathway. Reproduced with permission from Ref. [127]

Besides RP perovskites, Dion–Jacobson (DJ) perovskites form slabs that exactly on top of each other and connected by divalent (2+) interlayer spacers. Unlike RP perovskite phases (1/2, 1/2 displacements), DJ perovskite phases yield an eclipsed stacking arrangement (0, 0 displacements) that weakens the quantum confinement. It is believed that low-dimensional DJ perovskites have good structural stability and excellent carrier transmission performance [106, 123, 124]. Padture et al*.* reported a new type of DJ Sn halide low-dimensional perovskite based on ligand 4-(aminomethyl)piperidinium (4AMP), i.e., (4AMP)(FA)_n−1_Sn_n_I_3n+1_. PSC fabricated with (4AMP)(FA)_3_Sn_4_I_13_ obtained a PCE of 4.22%. The unencapsulated device was exposed to 1 sun illumination in N_2_ atmosphere at 45 °C for 100 h and only lost 9% of initial PCE. They summarized that compared to the RP phases bonded by relatively weaker van der Waals bonding; DJ phases bonded by stronger interlayer bonding would show enhanced stability. Meanwhile, photocarrier transport could be improved due to the divalent organic spacers that reduce the overall organic content [125]. Meanwhile, based on powder XRD patterns of (4AMP)SnI_4_, the interlayer spacing of adjacent Sn-I layers is calculated to be 10.4 Å, which is beneficial for the carrier transmission [126]. Later, Song et al*.* studied low-dimensional DJ phase perovskites by incorporating 1,4-butanediamine (BEA) into FASnI_3_. As shown in Fig. 9a, the interlayer of perovskite slabs is calculated to be 3.25 Å. The short distance weakens the quantum confinement and improved the stability by the strong interaction between the neighboring layers. Transient absorption (TA) spectra in Fig. 9b showed distinct bleach peaks at 610, 715, and 780 nm, representing *n* = 1, 2, and 3 perovskite phases. Ultrafast TA in Fig. 9c showed that excitons are formed in *n* = 1 (610 nm), *n* = 2 (720 nm), and *n* = 3 (780 nm) perovskite phases instantaneously. After the fast build-up, the photogenerated excitons from *n* = 1, *n* = 2, and *n* = 3 phases would localize to 3D-like phases within 0.36 ps, revealing that compact (BEA)FA_2_Sn_3_I_10_ film had weakened quantum confinement with improved carrier diffusion and mobility (Fig. 9d). The relevant PCE of the PSC reached 6.43%, accompanied with better stability against humidity and thermal corrosion than the FASnI_3_ devices [127].

### Ligands for Forming Heterojunction Structure

**Fig. 10:**
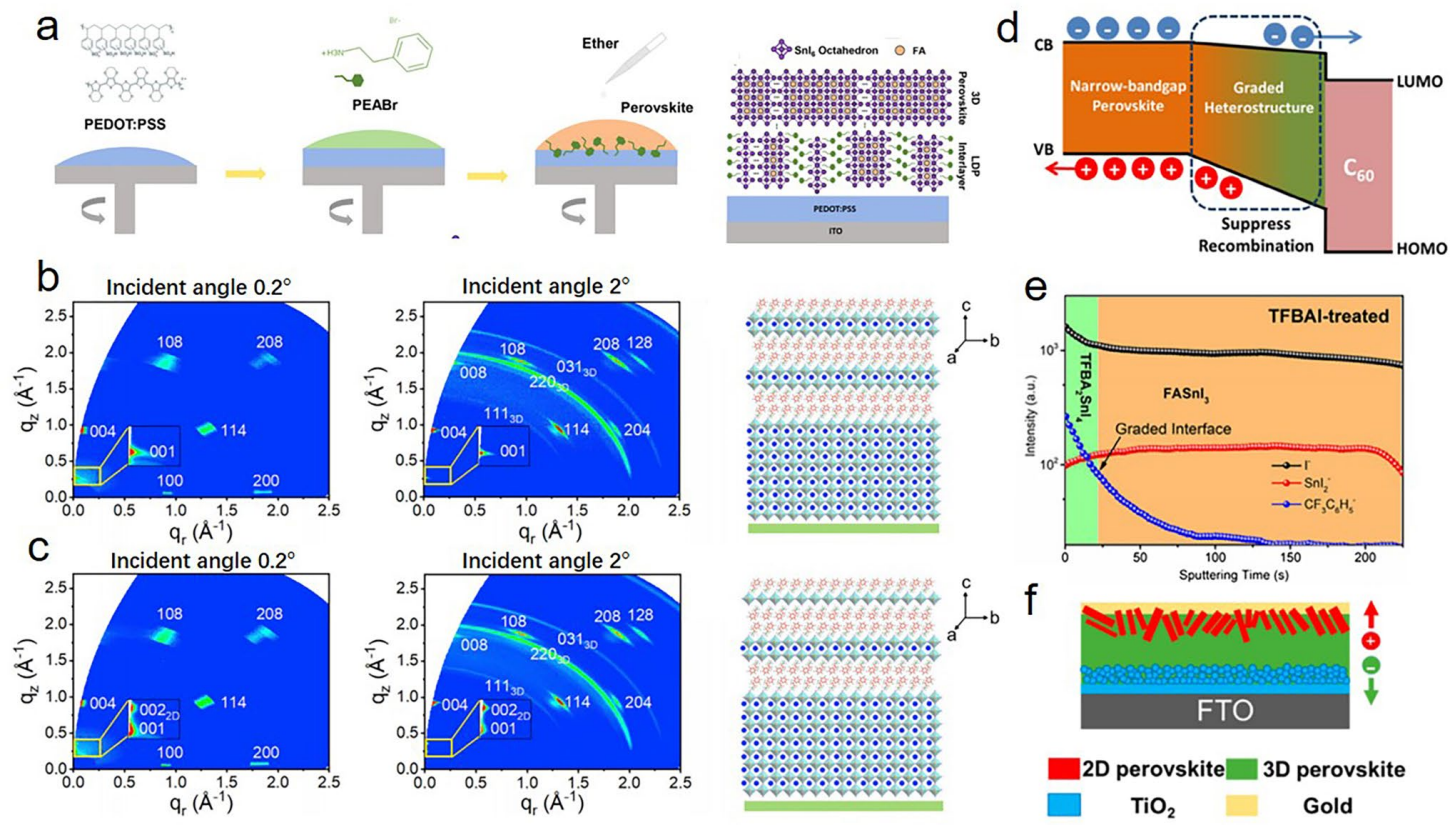
**a** Schematic diagrams for the introduction of LDP interlayer and schematic illustration of the LDP at the interface. Reproduced with permission from Ref. [128]. **b** GIWAXS images of the control film with incident angle of 0.2$^\circ$ and 2$^\circ$, and schematic structure. **c** GIWAXS images of HSP with incident angle of 0.2$^\circ$ and 2$^\circ$, and schematic structure. Reproduced with permission from Ref. [130]. **d** The schematic band alignment of the GHS at perovskite/fullerene interface. **e** ToF–SIMS depth profiles scanning from the top to the bottom of TFBAI-treated film. Reproduced with permission from Ref. [132]. **f** Proposed architecture of the HTM-free TEA 2D/3D PSC. Reproduced with permission from Ref. [133]

Gong and co-workers utilized PEABr ligand to introduce an ultrathin low-dimensional perovskite (LDP) interlayer close to the PEDOT:PSS/perovskite interface. The interlayer was achieved by spin-coating PEABr solutions onto the PEDOT:PSS layer, followed by the deposition of perovskite precursor solution (as illustrated in Fig. 10a). In the XRD pattern of PEABr incorporated FASnI_3_ film, an emerging $\left( {10\overline{1}} \right)\prime$ peak was exhibited, indicating the lattice distortion due to the formation of LDP. Besides, a reflection at ~ 5.8$^\circ$ was observed, supporting the 2D nature of the formed perovskite. SEM images showed the improvement of perovskite film morphology with PEABr ligand assisted interlayer, giving proof of the presence of LDP and its ability of assisting the growth of bulk perovskites. Next, they fabricated the PSC with a structure of ITO/PEDOT:PSS/FASnI_3_/C_60_/BCP/Cu. The champion PCE reached 7.05%, with a *V*_*oc*_ of 0.45 V, a *J*_*sc*_ of 24.87 mA cm^−2^ and an FF of 63%. Such improvement was due to the LDP interlayer could effectively passivate hole traps and reduce the charge recombination, increasing the charge carrier extraction efficiency at the interface [128]. Contrary to interlayer at PEDOT:PSS/perovskite interface, He et al*.* introduced a low-dimensional perovskite layer by spin-coating PEABr on the surface of pristine FASnI_3_ film. XPS etching spectra and ToF–SIMS confirmed the existence of PEABr containing low-dimensional layer at the surface of the perovskite film. The existence of such layer could help suppress Sn^2+^ oxidation, improve crystallinity and form a better match of electronic structure with hole- and electron-transporting layer materials [129].

Ning et al*.* found that the incorporation of PEAI ligand would help to form quasi-2D perovskite on top of the Sn-based perovskite film. Further, they incorporated pseudo-halogen ammonium thiocyanate (NH_4_SCN) in Sn-based perovskite to manipulate the crystal growth process. Characterization results indicated that a 2D-quasi-2D-3D hierarchy structure perovskite (HSP) structure was formed. XRD pattern showed that with the inclusion of 5% NH_4_SCN, two additional peaks at angles of 5.5$^\circ$ and 27.4$^\circ$, representing the crystallographic planes (200) and (1000) of 2D PEA_2_SnI_4_ appeared. Furthermore, GIWAXS was exploited to characterize the structure of PEAI-incorporated Sn-based perovskite. In Fig. 10b, c, when the incident angle was 0.2$^\circ$, the presence of (001) and (004) Bragg spots indicated the structure of quasi-2D perovskite (PEA_2_FASn_2_I_7_) on the surface of both films that with and without NH_4_SCN. Meanwhile, the (002)_2D_ spot above (001) in Fig. 10c could be ascribed to a single layer perovskite of 2D PEA_2_SnI_4_. As the incident angle increased to 2$^\circ$, emerged three Debye–Scherrer rings indicated 3D perovskite grains with random orientation deep in the film. They then fabricated solar cells based on 2D-quasi-2D-3D hierarchy perovskite structure with an inverted structure, utilizing NiO_x_ as hole-transporting layer and PCBM as electron-transporting layer. 5% addition of NH_4_SCN led the PCE of Sn-based PSC up to 9.41%, with a *V*_*oc*_ of 0.61 V, a *J*_*sc*_ of 22.0 mA cm^−2^ and an FF of 70.1%. Moreover, the unencapsulated devices was stable in duration as long as above 600 h. Such improvement should be due to the suppressed oxidation induced by 2D perovskite layer on the top of perovskite films [130]. Stranks et al*.* demonstrated Pb–Sn perovskite heterostructures formed between low-bandgap 3D and higher-bandgap 2D components by introducing a precursor solution of nominal PEA_2_FA_2_(Pb_0.5_Sn_0.5_)_3_I_10_ (*n* = 3) composition and NH_4_SCN additive. They revealed that the 2D domains formed preferentially on the surface of the films and stabilized the film properties [131].

Han et al*.* built a graded heterostructure (GHS) of perovskite light-absorbing layer to selectively extract the photogenerated charge carriers at the perovskite/electron transport layer interface. To fabricate the GHS of the Sn-based perovskite, the as-prepared FASnI_3_ film was dipped in a chloroform solution containing 4-(trifluoromethyl)benzyl ammonium iodide (TFBAI) salts, followed by thermal annealing to promote the sequential exchange reaction between FA^+^ and TFBA^+^ cations. It was found that low-dimensional perovskite TFBA_2_SnI_4_ with wide bandgap on top of the perovskite film was formed. Due to the large steric effect of bulky TFBA^+^ cation, the further reaction inside the perovskite crystal could be hindered, and a structure of (TFBA_2_SnI_4_)_x_(FASnI_3_)_1−x_ with graded bandgap alignment was supposed to be constructed (Fig. 10d). ToF–SIMS was used to characterize the depth of corresponding elements. As shown in Fig. 10e, the tin and iodine elements exhibited a homogeneous distribution throughout the control film, while the TFBAI-treated sample depicted a gradual decay of TFBA^+^ cations with increasing probed depth. As a result, the PSC with TFBAI treatment showed a PCE of 10.96% with stable power output. The graded structure of GHS perovskite was believed better for the charge separation and extraction at the perovskite/charge transport layer interface. Meanwhile, TFBA ammonium molecule could significantly reduce the trap density in perovskite films due to its passivation effect [132].

An HTM-free configuration in n-i-p structure was studied by Chen et al*.* by employing thienylethylammonium (TEA) to form 2D perovskite at the top of perovskite film. As illustrated in Fig. 10f where the red rods and green area represent the well-aligned 2D and 3D perovskites, such a proposed 2D/3D configuration realizes the capability of generating a p-n-like junction and hence efficient charge separation to boost the performance of HTM-free Sn-based PSCs. Therefore, the band positions of 3D perovskite and 2D perovskite line up well for the charge separation. The relevant Sn-based PSC achieved a PCE of 5.17%, which was a remarkable value reported in HTM-free Sn PSCs [133].

Lately, Yan et al*.* developed a quasi-2D(down)/3D(top) stratified vertical heterojunction structure via vacuum treatment after film coating, while the application of guanidinium thiocyanate (GuaSCN) tuned the electronic properties in the heterojunction as an additive. It was speculated that the organic solvent would quick evaporate from the top of surface, and thus making less soluble 3D perovskite solidified on the top while the 2D phases with higher solubility aggregated at the bottom. Such heterojunction was beneficial for the carrier separation and transfer across the junction. Furthermore, GuaSCN helped in building conducting channels for hole transportation in 2D layer, as well as suppressing trap-assisted recombination loss in the film. As a result, a Sn-based PSC with NiO_x_ as HTL and ICBA as ETL achieved a PCE of 13.79% and an open circuit voltage as high as 1.01 V, which is the highest value reported by now [134].

## Effect of Ligands on Stability

In this section, compositional engineering aims at structural stability by tuning different chemical ligand combinations at X-site (I^−^, Br^−^, Cl^−^, etc.) and A-site (FA^+^, MA^+^, Cs^+^, etc.) is discussed. Moreover, the detrimental self-doping caused by the existence of Sn^4+^ in the perovskite film, along with the degradation resulted by oxygen and moisture, is considered to be the stability bottleneck of Sn-based PSCs. Thus, the strategies of post-treatment will be introduced to de-dope the surface Sn^4+^ defects and protect the perovskite film from oxygen and moisture.

### Compositional Engineering at X- and A-site

Compositional engineering has been widely proved to be an effective method to enhance the properties of perovskites and optimize the performance of relevant perovskite solar cells. The mixing of monovalent alkali cations and halide anions is one of the most widely utilized methods in the composition engineering of Pb- and Pb/Sn-based perovskites [135–137]. For Sn-based cases, FASnI_3_ owns a larger resistance to oxidation than MASnI_3_, and has been widely investigated as a typical Pb-free perovskite. Nevertheless, the tolerance factor (*t*) of FASnI_3_ is 1.04, which is due to the large radius of FA^+^ cation and causes phases instability and poor crystallinity, so exploring methods of mixing FA^+^ cation with other monovalent cations (e.g., MA^+^ and Cs^+^ cations) deserve more attention. The FA_1−x_MA_x_SnBr_3_ system is proved to possess a cubic symmetry with lattice parameter and cell volume obeying Vegard's law. A wide tuning of the band gap from 2.4 to 1.9 eV would induce by the FA/MA substitution, which could originate from the contribution of FA and/or MA to the density of defects and in turn to the valence band characteristics [138]. Meanwhile, it has been extensively acknowledged that a partial change of the halide composition in the lattice of Pb-based halide perovskites would bring synergetic effects on the material. For example, a partial mixing of Br^−^ ions into MA(or FA)PbI_3−x_Br_x_ would change the crystal structure from tetragonal to cubic and control the band gaps simultaneously [139, 140]. In addition, it could be expected that the mixing of specific halide anions would lead to optimized stability against humidity.

#### X-site Mixing

**Fig. 11:**
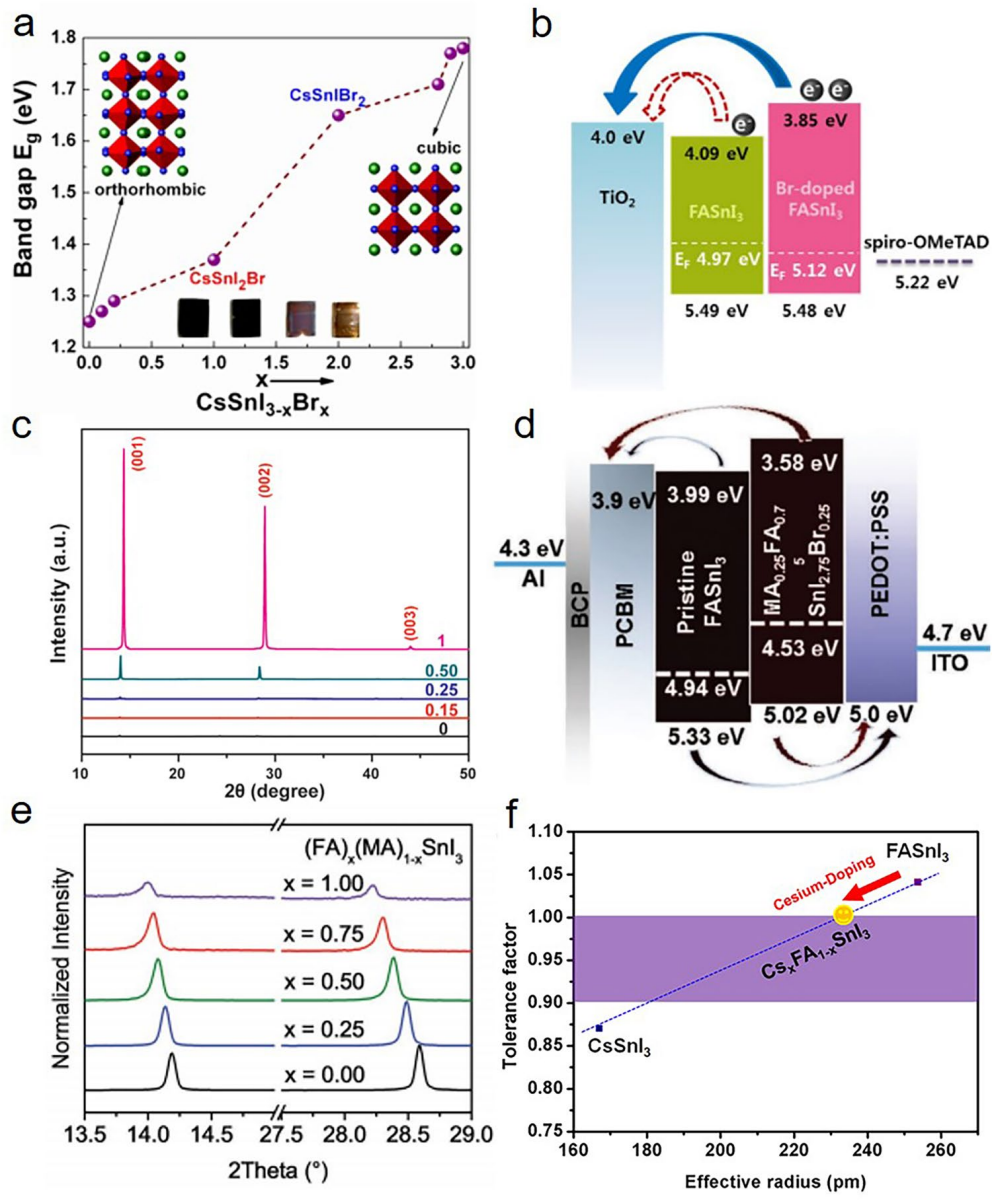
**a** Bang gap variation with respect to Br.^−^ concentration. The inset shows the photographs of samples: CsSnI_3_, CsSnI_2_Br, CsSnIBr_2_, and CsSnBr_3_ from left to right. Reproduced with permission from Ref. [141]. **b** Schematic energy diagram of TiO_2_, FASnI_3_, and Br-doped FASnI_3_ films. Reproduced with permission from Ref. [143]. **c** XRD patterns for MA_x_FA_1−x_SnI_3−x_Br_x_ perovskites (*x* = 0, 0.15, 0.25, 0.5, 1). **d** Energy band alignment of the inverted planar PSCs containing both FASnI_3_ film and MA_0.25_FA_0.75_SnI_2.75_Br_0.25_ perovskite films. Reproduced with permission from Ref. [144]. **e** XRD patterns of FA_x_MA_1−x_SnI_3_ (*x* = 0.00, 0.25, 0.50, 0.75, and 1.00) films in the region 13.5°–29.0°. Reproduced with permission from Ref. [147]. **f** Correlation between the tolerance factor and the effective radius of Cs/FA cation in Cs_x_FA_1−x_SnI_3_ perovskite. Reproduced with permission from Ref. [154]

Mathews et al*.*, early in 2014, studied the impact of Br^−^ doping into CsSnI_3_ perovskite. The bandgap of perovskite increased from 1.27 eV for CsSnI_3_ to 1.37, 1.65, and 1.75 eV for CsSnI_2_Br, CsSnIBr_2_, and CsSnBr_3_, respectively. As shown in Fig. 11a, as the proportion of Br^−^ doping increases, the color of the perovskite film gradually turns from black to dark brown and then to light brown, which will reduce the light harvesting while benefitting the increase of open circuit voltage. The addition of Br^−^ resulted in the obvious reduction of Sn cation vacancies and structural disorder [141]. Later, Diau et al*.* managed to synthesis and characterize tri-halide mixed tin perovskites (MASnIBr_2−x_Cl_x_). XRD confirmed that when the SnCl_2_ proportion was $\ge$ 50% (*x* $\ge$ 1), phase separation would occur to produce MASnI_3−y_Br_y_ and MASnCl_3−z_Br_z_. After optimization, the PSCs based on MASnIBr_1.8_Cl_0.2_ exhibited the best J–V performance and long-term stability. The corresponding PSC fabricated with the carbon-based mesoscopic structure and free of hole-transporting layers achieved a PCE of 3.1%. The small proportion of Cl inside the Sn-based perovskite crystals would effectively suppress the charge recombination, decrease the charge accumulation, and prolong the carrier life time [142].

Likewise, Seo et al*.* also investigated the optimization of FASnI_3_ perovskite by introducing Br anion. With the substitute of larger I atoms with smaller Br atoms in the FASnI_3_ lattice, the diffraction peaks of XRD patterns showed a gradual shift toward higher degrees, in agreement with the reduction of the lattice spacing. The band gap of the perovskite films was widened while the conduction band edge was lifted to a higher level with increasing amounts of Br, as illustrated in Fig. 11b, thus facilitating the electron transfer into TiO_2_ due to more suitable energy-level matching. Moreover, Br-doping played a key role in reducing the defect concentration and hence decrease the carrier density of the perovskite material. As a result, the Br-doping FASnI_3_-based PSC gained the PCE of 5.5% with remarkable photostability for encapsulated device [143]. The function of Br anion in FASnI_3_ perovskite was also studied by He and co-workers. They mixed MABr into FASnI_3_ precursor solutions to fabricate MA_x_FA_1−x_SnI_3−x_Br_x_ perovskites (*x* = 0, 0.15, 0.25, 0.5, 1). With the increase of MABr in the composition, the peak intensities in XRD pattern representing (001) series were enhanced obviously (Fig. 11c). Especially when *x* = 1, only peaks representing (001), (002), and (003) were found in XRD patterns, indicating high orientation growth and crystallinity of MABr-mixed perovskite films. It could also be observed from the absorption spectra that the absorption edges gained a blue shift with the alloying of MA^+^ and Br^−^, which are in consistent with the aforementioned literature. The relevant electronic structures of FASnI_3_ and MA_0.25_FA_0.75_SnI_2.75_Br_0.25_ perovskite films are revealed based on UPS measurement and shown in Fig. 11d. MABr-mixed perovskite exhibited a better match with both HTL (PEDOT:PSS) and ETL (PC_61_BM) than the pristine film, and herein gained stronger carrier transportability at the bi-interfaces, resulting in the improved device performance. Based on such investigation, the PCE of the MA_0.25_FA_0.75_SnI_2.75_Br_0.25_-based PSC reached 9.31%, in contrast to 5.02% of the control FASnI_3_ device [144].

Besides halide anions, the incorporation of pseudo-halogen, such as [BH_4_]^−^ and [AlH_4_]^−^, was proved to be beneficial for realizing the enhancement of oxidation resistance of Sn^2+^ in MASnI_3_ perovskites because of the large electron transfer between Sn^2+^ and [BH_4_]^−^ and [AlH_4_]^−^. Meanwhile, in MASnI_2_BH_4_ and MASnI_2_AlH_4_ perovskites, high carrier mobility could still be preserved and only a slight decrease in optical absorption strength was observed [145]. Based on theoretically investigation, Diau et al*.* synthesized pseudo-halogen-based tin perovskite FASnI_3−x_(BF_4_)_x_, and fabricated PSCs with mesoscopic carbon-electrode architecture. According to XRD characterization and plane-wave DFT calculations, the structural integrity of this kind of perovskite was maintained. Meanwhile, a red shift in the PL spectra was relative to an upward shift of the VBM by the replacement of I^−^ with BF_4_^−^. It could be concluded that the rapid charge transfer and decreased recombination and background carrier density resulted in the enhancement of the corresponding carbon-electrode PSCs. The FASnI(BF_4_)_2_-based PSC exhibited a PCE of 1.3% with better stability under light soaking and dark storage conditions [146].

#### A-site Mixing

The composite perovskite FA_0.75_MA_0.25_SnI_3_ was introduced by Huang et al*.* in an inverted PSC structure. Firstly, XRD patterns of FA_x_MA_1−x_SnI_3_ (*x* = 0.00, 0.25, 0.50, 0.75, and 1.00) films were measured and shown in Fig. 11e. The main peaks located at around 14$^\circ$ and 28$^\circ$ could be ascribed to the (101) and (202) lattice planes. Only one peak for each lattice planes of the mixed A-site perovskites was observed, indicating that FA and MA cations were evenly distributed in the perovskite lattice rather than forming phases of different species. Moreover, the diffraction peaks for (101) and (202) shifted to a higher angle with the increase of MA content, suggesting the expansion of lattice parameters, which could be due to the gradual replacement of the larger FA cations by smaller MA cations. In terms of optical properties, the band gaps calculated by steady-state PL spectra exhibited a decrease trend as the MA content increase. To further elucidate the impact of A-site cation mixing, PSCs were fabricated with PEDOT:PSS as HTL and C_60_ as ETL. Among all the proportions of MA and FA cations, FA_0.75_MA_0.25_SnI_3_-based device gained a champion PCE of 8.12%, which was superior to the champion PCE of 4.29% for MASnI_3_-based and 6.60% for FASnI_3_-based ones [147]. Similarly, FA_0.75_MA_0.25_SnI_3_ perovskite was used to study the influence of antisolvent diethyl ether (DE), toluene (TL), and chlorobenzene (CB), respectively. The results showed that antisolvent CB could lead to a dense and uniform Sn-based perovskite film [148]. Meanwhile, the FA-MA-mixed perovskite with the same proportion was also investigated by Jo et al*.* on the FTO/Blocking TiO_2_/Mesoporous TiO_2_ substrate. The incorporation of FA in MASnI_3_ would improve the crystallinity and red shift the absorption edges measured from UV–vis absorption spectra. They used Kelvin probe force microscopy (KPFM) to measure the surface photovoltage (SPV) spectroscopy, and mesoporous TiO_2_ showed significant changes in the electronic structure and built-in potentials at the interfaces with FA_0.75_MA_0.25_SnI_3_, which was beneficial for the charge carrier transfer at the perovskite/ETL interface [149].

Besides MA^+^ and FA^+^, organic cation guanidinium (C(NH_2_)_3_^+^, GA^+^), which has zero electric-dipolar moment and slightly larger size ($\approx$ 278 pm) than that of FA^+^ ($\approx$ 253 nm), with the empirical Gold-Schmidt tolerance factor of GASnI_3_ being 1.051, might be a suitable A-site candidate for Sn-based PSCs [103, 150, 151]. Diau et al*.* managed to mix GAI with FAI in varied proportions and tested relating performance. With the increasing of GAI proportion, the size of perovskite crystal increased due to the larger cation size, but phase transition was not observed. Meanwhile, it could be confirmed from the XRD pattern that GA^+^ cations inserted into the 3D perovskite lattice structure, adopting the same structure as FASnI_3_ with an orthorhombic unit cell, space group *Amm2*. With the introduction of GAI, the PL lifetime also increased, indicating fewer defect states with particular proportions of GAI. According to UPS measurement, the presence of GA^+^ cation could alter the electronic structure of perovskite and shift the VB level to achieve a better match of the neighboring hole-transport layer (PEDOT:PSS). The corresponding PSCs were fabricated and the champion device showed a PCE of 9.6% with 20% GAI incorporation [152]. The effect of mixing guanidium cation at A-site was also investigated by Saeki and co-workers. Time-resolved microwave conductivity (TRMC) measurement was carried out to provide insight into the charge carrier dynamics. A ternary A-site cation-mixed Sn-based perovskite (GA_x_FA_1−x_)_0.9_PEA_0.1_SnI_3_ (*x* = 0–1) gained the passivated grain surface and improved TRMC electron mobility (${\mu }_{e}$) when ratios of GA cations were in the range of 0–0.25. Accordingly, the relevant PSC exhibited the maximum PCE of 7.90% at *x* = 0.15 [153].

Alkali cation-doping was also believed to be an effective way to reduce the tolerance factor of FASnI_3_ (1.04) due to the large radius of FA^+^ cation, and thus optimizing the phase stability and crystallinity. Wu et al*.* proposed a structural regulation strategy to regulate the geometric symmetry of FASnI_3_ by Cs cation mixing. The mixing of FA with Cs at A-site could make the tolerance factor downward from 1.04 to 1, which would form the ideal high-symmetry cubic structure (Fig. 11f). This incorporation resulted in an enhanced UV–vis absorption and red-shifted PL spectra as compared with pristine FASnI_3_ film. Meanwhile, DFT calculations revealed the enhanced thermodynamic stability with the increased proportion of Cs. The fabricated PSCs with inverted device structure achieved the champion PCE of 6.08% with 8% Cs incorporation and impressive stability in N_2_ and in air atmosphere [154]. The role of Rb mixing in A-site has also been investigated. Hatton et al*.* studied the properties of 3D perovskite Cs_1−x_Rb_x_SnI_3_, the result suggested that the small amount of Rb incorporation (*x* = 0.2) could promote the performance of PSC with sufficient stability and light harvesting capability [155]. Similarly, Miyano et al*.* explored the effects of Rb insertion in the FASnI_3_ lattice. XRD patterns for FA_1−x_Rb_x_SnI_3_ films showed that the small crystallite size was formed with higher Rb content. They also found that when *x* = 0.08, a highly covered Sn-based perovskite film with significantly suppressed defect density (from ~ 1.86 $\times$ 10^17^ cm^−3^ for FASnI_3_ to ~ 2.86 $\times$ 10^16^ cm^−3^ for FA_0.92_Rb_0.08_SnI_3_) was obtained. As a result, the Sn-based PSC showed a PCE of 5.89% and the encapsulated device showed improved stability for over 20 days in ambient air [156].

### Post-treatment for Improving Stability

Post-treatment for passivating surface defects and improving stability has been widely studied in Pb-based perovskites. For Sn-based perovskite, however, the investigation is limited due to the relatively high solubility in IPA solvent. Therefore, some reported methods of post-treatment aiming at improving Sn-based perovskite stability are concluded below.

The fabrication of a semiconducting-insulating interface to fully cover the Sn-based perovskite film with a thin layer of poly(methylmethacrylate) (PMMA) (dissolved in CB solution) was introduced by Yamauchi and co-workers. PMMA offered a layer of protection from oxygen and moisture, which caused by the enhanced hydrophobicity. Meanwhile, the thin layer also passivated the defect-driven recombination, limited the penetration of oxygen inside the perovskite layer. The PMMA-modified PSC retained ~ 80% of its initial PCE after 240 h of shortage under ambient conditions (25 °C, 60% RH), while the control device dropped to ~ 1% PCE within 3 days [157]. Chen et al*.* reported a post-treatment of Sn-based perovskite film by a bi-functional thin layer of ethylenediamine formate (EDAFa_2_) (dissolved in CB) to simultaneously passivate the interfacial defect and improve the stability of Sn^2+^. A thermodynamically stable chemical environment was created due to the strong coordination bond between EDAFa_2_ and Sn^2+^; thus, the grain encapsulation would stabilize the perovskite structure. The UPS measurement showed a better energy-level alignment between perovskite layer and C_60_ ETL after EDAFa_2_ post-treatment, which resulted in a promoted electron transfer. The relevant unencapsulated interfacial-modified PSC device retained ~ 95% of initial PCE after 1960 h of storage in N_2_ environment [158].

Diau et al*.* treated the FA_0.8_GA_0.2_SnI_3_ perovskite film with phenyl-hydrazinium thiocyanate (PHSCN) dissolved in solvent 2,2,2-trifluoroethanol (TFE), where the PH cation could act as a reducing agent for surface passivation and pseudo-halide SCN^−^ anion could partly replace I^−^ anion for surface protection. The investigation showed that the post-treatment of PHSCN effectively passivate the surface of perovskite surface and improve the electron transfer from perovskite layer to C_60_ ETL. Furthermore, the performance of PHSCN modified PSC device gained a gradual improvement during storage, for which the best efficiency (13.5%) was obtained after stored for 1272 h in N_2_-filled glovebox; the device also retained 92% of maximum PCE after 3000 h storage [159]. A post-treatment of a hydrophobic bulky molecule of 3-(trifluoromethyl) phenethylamine hydroiodide (CF_3_PEAI) was carried out by Hao et al*.* recently. The solute was dissolved in a mixed solvent of 1,1,1,3,3,3-hexafluoro-2-propanol (HFP):CB = 1:4 (volume/volume). This interlayer suppressed the interfacial non-radiative recombination and thus extended the carrier lifetime. Moreover, the steady-state PL spectra of perovskite film with CF_3_PEAI modification showed a peak at wavelength of 613 nm, which belonged to the 2D (CF_3_PEA)_2_SnI_4_ perovskite; such 2D interlayer could further reduce the interfacial voltage loss. The relevant Sn-based PSC gained a PCE of 10.35% and maintained over 80% after a storage of over 1700 h in N_2_ condition; Meanwhile, the modified device still exhibited about 70% of initial PCE after kept in air (20 °C, 15% RH) for 150 h, which was believed to be the result of defect passivation, hydrophobicity increase, and crystal structure stabilization [160].

To regulate the crystal growth, Huang et al*.* proposed a seeded growth (SG) method for post-treatment. One layer of FASnI_3_ perovskite was first deposited by the typical solution method, then one more layer of the same perovskite film was spin-coated in the same way. The precoated layer would serve as a seed layer and regulate the crystallization of Sn-based perovskite grains. Therefore, PSCs treated by seeded growth (SG) method maintained ~ 61% of initial PCE after storing in ambient condition (30–50% RH) after 24 h, which could result from the larger grain size and fewer grain boundaries after SG treatment [161]. Hayase et al*.* showed their investigation on vapor-assisted surface passivation. The perovskite film was placed under an inverted petri dish filled with the solution dissolved with passivation molecule. They compared three kind of passivation molecules: ethane-1,2-diamine (EDA), bromotrimethylsilane (Me_3_SiBr), acetylacetone (ACAC) that dissolved in CB, respectively. The result showed that vapor passivation provided more optimized film morphology and relevant device *J–V* performance than liquid passivation. The EDA-vapor passivated device maintained 85% of initial PCE after stored 40 days in N_2_-filled glovebox [162].

**Fig. 12:**
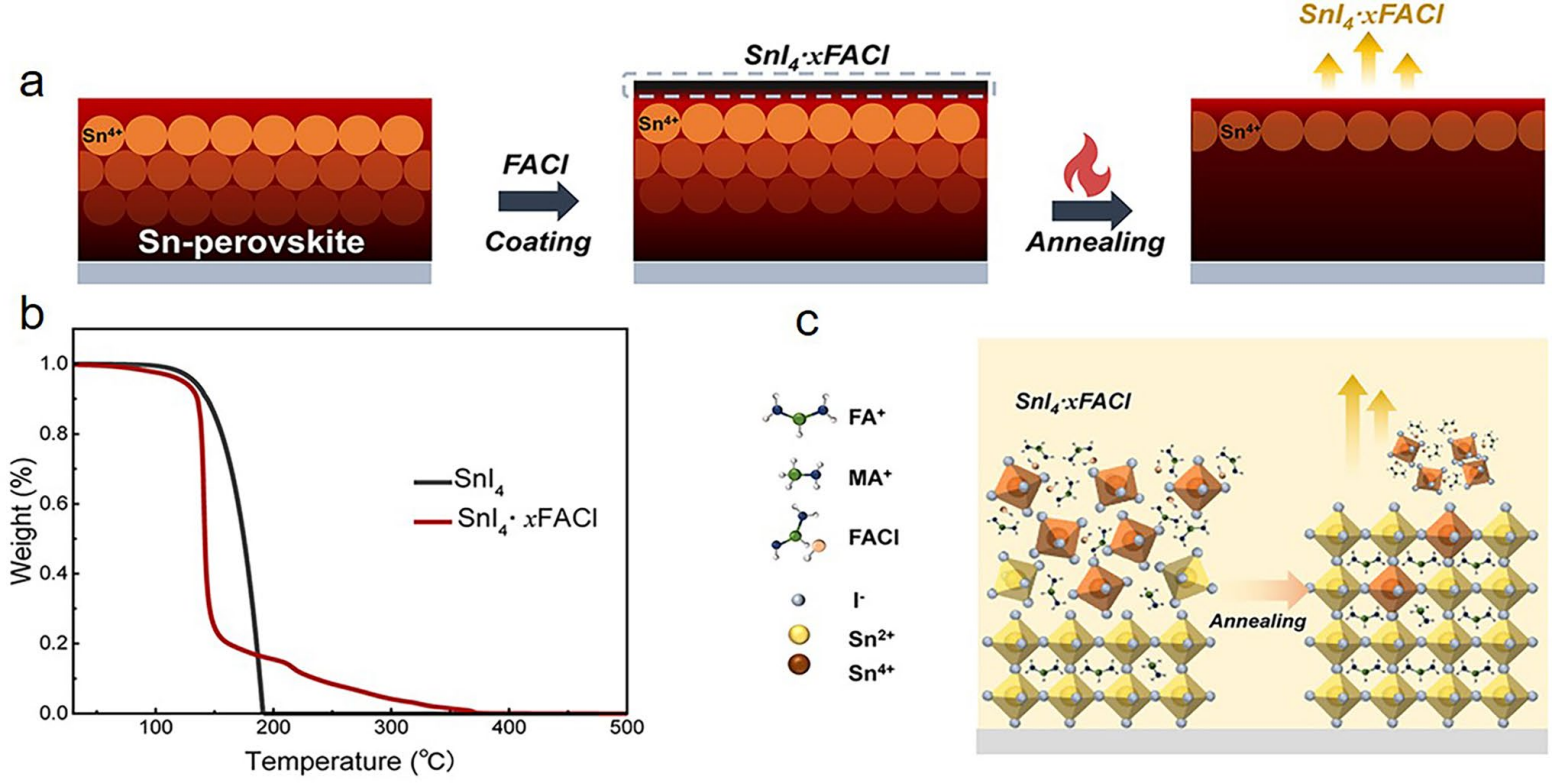
**a** Schematic description of the adopted chemical method for surface dedoping of Sn perovskite films. **b** TGA analyses of SnI_4_ and SnI_4_$\cdot$xFACl. **c** Schematic illustration of the surface dedoping of Sn perovskite films induced by SnI_4_$\cdot$xFACl complexation and volatilization. Reproduced with permission from Ref. [16]

The halide-based additives have been widely studied in Pb-based PSCs [163, 164], Nevertheless, He et al*.* pointed out that additive added directly into the precursor solution usually led to uncontrollable nucleation sites, which limited the optimization of grain growth and crystallinity. Therefore, post-treatment strategies using halide-based additives need to be developed, which have rarely employed in the field of Sn-based perovskites. They studied the secondary-crystallization growth (SCG) process by dissolving chloride-based molecules (MACl and FACl) in isopropyl alcohol and spin-coating onto perovskite films. The post-treatment of amine chlorides contributed to the increase of grain size, suppression of Sn^2+^ oxidation, reduction of trap state, as well as the improvement of hole transport mobility. Furthermore, a more matched energy level with adjacent carrier transporting layers was obtained after SCG process. The encapsulated devices are stored in N_2_-filled glovebox, the SCG device maintained 87% initial PCE after 1000 h storage [165]. Recently, Zhou et al*.* demonstrated a surface dedoping approach to remove Sn(IV) self-dopants that mainly accumulated on the surface of Sn-based perovskite films to optimize the device stability (schematically illustrated in Fig. 12a). A thin layer of FACl was deposited onto FA_0.75_MA_0.25_SnI_3_ film through thermal evaporation. They confirmed that a coordination complex of SnI_4_$\cdot$xFACl was formed on the surface of perovskite film. By analyzing thermogravimetric (TGA) results in Fig. 12b, they found that SnI_4_$\cdot$xFACl could be volatilized at 60 °C, which was a relatively low temperature compared with SnI_4_ (115 °C). This difference might relate to the varied bonding nature caused by the organic–inorganic complexation. In this case, the removal of SnI_4_$\cdot$xFACl complex through a sequential thermal annealing means the simultaneous removal of Sn(IV) self-dopants. A schematic illustration of related chemistries in this chemo-thermal surface dedoping process is depicted in Fig. 12c. XPS measurement confirmed the overall decrease of Sn(IV) at all depth of perovskite film. As a result, the relevant Sn-based PSC showed a PCE of 14.7%, as well as a remarkable stability for retaining 92% initial PCE after a storage for over 1000 h in N_2_-filled glovebox [16].

## Conclusion and Prospect

Tin halide perovskites have been recognized as promising materials for environment friendly photovoltaic devices. With global-wide efforts, the reported record PCE has been close to 15%. This value, however, is still much lower than the theoretical efficiency of 33.4% for those photovoltaic materials with bandgap of 1.4 eV. This situation implies that the optimization of Sn-based perovskite layer is still the core problems to be solved. Ligand engineering, due to its flexible customizability, is considered as the powerful strategy for improving the performance of Sn-based perovskites, is systematically discussed here and classified according to its course of action: (1) the source stage, including antioxidant that added into the precursor solution to prevent Sn^2+^ oxidation; (2) the intermediate fabrication state, including ligands that help to form perovskite films with highly orientated crystallization and improved morphology, or alternatively, to form low-dimensional structures that benefit the charge carrier transportation; and (3) the after-preparation state, aiming at the improvement of the stability of Sn-based PSCs, where the compositional engineering to adjust structural stability and post-treatment engineering to passivate surface defects are introduced.

On the other hand, ligand engineering for Sn-based perovskites could also be classified according to their unique features and functional groups: (1) the coordination with Sn^2+^ cations in perovskite or SnX_2_ additive to prevent oxidation, including ligands with functional groups of lone pair electrons (carbonyl groups, amide groups, ether groups, etc.); (2) the coordination with halide anions to optimize the crystallization, including ligands with amino groups and hydroxyl groups, etc. Meanwhile, according to the volume and doping ratio, ligands can assist to passivate bulk defects or form low-dimensional structure; and (3) the compositional modification to improve stability at A-site, including FA^+^, MA^+^, GA^+^, Cs^+^, Rb^+^, or alternatively, at X-site, including I^−^, Br^−^, Cl^−^, and pesudohalogen.

Based on all the merits discussed of ligand engineering throughout each fabrication stage, in-depth studies on ligand profiles are foreseen in order to further improve the photovoltaic performance of Sn-based PSCs:

1. The susceptibility of Sn^2+^ to oxidation remains a central challenge limiting PCE enhancement: i) Efforts need to be made to ensure the purity of SnI_2_ source, since most reported studies used SnI_2_ that directly purchased without purification. Some groups have showed the effectiveness of SnI_2_ source modification (the addition of Sn powder, one-step synthesis of SnI_2_$\cdot$(DMSO)_x_ adduct in precursor solution [57, 166], etc.), which might be an easily ignored point to improve device efficiency; ii) The irreversible oxidation of Sn^2+^ caused by DMSO solvent has been recently recognized, which forces researcher to find brand new solvents with high solubility of precursor chemicals, thermal stability, and possibility of forming perovskite, or alternatively, the use of ionic liquids as solvent may be a promising way to improve the reliability and stability during film formation.
2. The defects in the Sn-based perovskite film that result in crystal distortion and non-radiative recombination need to be suppressed by the further investigation of ligands. Multifunctional ligands that could simultaneously coordinate with I^−^ anions and Sn^2+^ cations, adjust the band structure, form an orientated crystallization, and reduce bulk defects would be ideal choices. Theoretical calculations may offer hints for researchers to find such a proper material.
3. The stability issue still hinders the way toward practical application. Although the reported stability has reached over 2000 h of storage in N_2_-filled glove box, it should be noticed that such result falls far behind the industry requirement. In addition, the operational stability under the MPPT for Sn–PSCs undergoes less research, implying a large room to improve the performance reliability. The suppression of Sn vacancy defects has been proved as an effective way to improve the stability; thus, ligand-assisted crystallization as well as post-treatment for perovskite films should be further studied in future.
4. The fabrication of large-area Sn-based PSCs is a topic that need to be solved in face of commercialization. Tracing back to the origin, the uncontrolled nucleation with the low formation energy is stressed especially for scaling up the fabrication. Ligand engineering could be a useful method to obtain films with less defects. Meanwhile, rare studies have reported methods for coating solution-based large-area Sn-based PSCs, which is necessary for achieving high-efficiency Sn-based perovskite modules.
5. Sn–Pb mixed perovskites are becoming popular as narrow bandgap (1.2–1.3 eV) light absorbers in single junction PSCs and all-perovskite tandem solar cells [167, 168]. One of the main challenges is still the Sn^2+^ oxidation and relevant defects. Ligand engineering in Sn–Pb mixed perovskites have also been widely studied, including SnX_2_ additive [169], coordination with I anions [170–172] or Sn cations [173, 174], formation of low-dimensional structure [175], improvement of stability by A- & X-site mixing [176, 177] or post-treatment [178], etc. The research on Sn-based perovskite and Sn–Pb mixed perovskite can be mutually referenced to solve the problem of commonality.

In conclusion, ligand-assisted strategies play a vital role in optimizing performance of Sn-based perovskites solar cells. We believe that with further study, more appropriate ligand engineering through the whole fabrication process will enable the preparation of Sn-based PSCs with higher efficiency and stability toward multiple practical applications.
